# Systems Genetics of Liver Fibrosis: Identification of Fibrogenic and Expression Quantitative Trait Loci in the BXD Murine Reference Population

**DOI:** 10.1371/journal.pone.0089279

**Published:** 2014-02-28

**Authors:** Rabea A. Hall, Roman Liebe, Katrin Hochrath, Andrey Kazakov, Rudi Alberts, Ulrich Laufs, Michael Böhm, Hans-Peter Fischer, Robert W. Williams, Klaus Schughart, Susanne N. Weber, Frank Lammert

**Affiliations:** 1 Department of Medicine II, Saarland University Medical Center, Homburg, Germany; 2 Department of Medicine III, Saarland University Medical Center, Homburg, Germany; 3 Department of Infection Genetics, Helmholtz Center for Infection Research, University of Veterinary Medicine Hannover and University of Tennessee Health Science Center, Braunschweig, Germany; 4 Department of Pathology, University Hospital Bonn, Bonn, Germany; 5 Department of Anatomy and Neurobiology, University of Tennessee, Memphis, Tennessee, United States of America; 6 Department of Medicine II, Section Molecular Hepatology, Medical Faculty Mannheim, Heidelberg University, Germany; Central China Normal University, China

## Abstract

The progression of liver fibrosis in response to chronic injury varies considerably among individual patients. The underlying genetics is highly complex due to large numbers of potential genes, environmental factors and cell types involved. Here, we provide the first toxicogenomic analysis of liver fibrosis induced by carbon tetrachloride in the murine ‘genetic reference panel’ of recombinant inbred BXD lines. Our aim was to define the core of risk genes and gene interaction networks that control fibrosis progression. Liver fibrosis phenotypes and gene expression profiles were determined in 35 BXD lines. Quantitative trait locus (QTL) analysis identified seven genomic loci influencing fibrosis phenotypes (pQTLs) with genome-wide significance on chromosomes 4, 5, 7, 12, and 17. Stepwise refinement was based on expression QTL mapping with stringent selection criteria, reducing the number of 1,351 candidate genes located in the pQTLs to a final list of 11 *cis*-regulated genes. Our findings demonstrate that the BXD reference population represents a powerful experimental resource for shortlisting the genes within a regulatory network that determine the liver's vulnerability to chronic injury.

## Introduction

Liver fibrosis is a common consequence of chronic injury. Inducing agents vary from hepatotoxins, metabolic disorders and autoimmune reactions to viral infections. A characteristic feature of the fibrotic response is the ongoing repair mechanism resulting in an excessive accumulation of extracellular matrix [1], [2]. Fibrosis may progress to liver cirrhosis, which is characterized by severe distortion of liver architecture and impaired function. Of note, in patients with an exposure to similar environmental risk factors, the progression of liver fibrosis varies markedly. Based on the rate of fibrosis progression, patients may be classified as ‘slow’ or ‘rapid fibrosers’ [3]. These interindividual differences have been attributed to environmental, but also to genetic (and epigenetic) factors [1], [4], [5]. Several fibrogenic gene variants have been identified, e.g. complement component 5 (*Hc*) [6], other chemoattractants such as the chemokine *CXCL9* [7] and the chemokine receptor *CXCR3* [8] or metabolic enzymes like the triglyceride hydrolase adiponutrin (*PNPLA3*) [9], [10]. Moreover, two recent genome-wide association studies [11], [12] identified a set of novel potential susceptibility genes for liver fibrosis, including *PNPLA3*, but no specific networks underlying fibrogenesis were reported. However, due to the large number of factors involved, the systematic identification of genetic determinants and networks affecting hepatic fibrosis remains a major challenge.

Systems genetics is a powerful method to dissect the underlying mechanisms of complex traits, including predisposing gene networks and environmental variants [13], [14]. The key experimental set-up is to make use of a genetic reference population. Here, we availed of the BXD set of recombinant inbred (RI) mouse lines, which are inbred progeny of F2 intercrosses of the inbred mouse strains C57BL/6J and DBA/2J [13], [15]. RI lines are especially suited as a mapping panel, since they form an immortalized set of isogenic lines [16], [17], and a large number of animals and phenotypes per genome can be analyzed under standardized experimental conditions, thus lowering environmental noise. This improves the yield of information for the detection of genetic loci linked to trait variation, known as quantitative trait locus (QTL) mapping [13], [15]. In previous studies we have demonstrated that the parental strains C57BL/6J and DBA/2J show significant phenotypic variation of key fibrogenic parameters and therefore differ in their fibrosis susceptibility [6], [7], [18]. Since these strains also vary in four million genetic sites across their genome [19], they provide the phenotypic and genetic diversity necessary for mapping studies in liver fibrosis. Furthermore, with more than 13,000 genetic markers and over 3,000 phenotypic records the BXD lines are one of the best-characterized murine reference panels [13], [20], [21].

Our aim was to determine new gene variants that affect hepatic fibrosis and to apply a systems genetics approach for the identification of gene networks that are critical for fibrosis phenotypes. Therefore, we characterized differences in fibrosis susceptibility of BXD lines after induction of liver fibrosis with carbon tetrachloride (CCl_4_) and generated toxicogenomic, hepatic expression profiles by microarray analyses. Afterwards, we associated the genetic variation in our population with transcript variation in order to identify determinants of gene expression in liver fibrosis. In addition to single QTL and gene-gene interaction studies, the combination with expression genetics provided novel insights into potential networks modifying hepatic fibrogenesis.

## Methods

### Animals and experimental design

C57BL/6J, DBA/2J, B6D2 F1 hybrids and BXD lines were obtained from The Jackson Laboratory (Bar Harbor, ME) or from Oak Ridge Laboratory (lines BXD43, BXD51, BXD61, BXD62, BXD65, BXD68, BXD69, BXD73, BXD75, BXD87, BXD90), and were bred in the facility of the Neurobsik consortium from the VU University Amsterdam. The mice were maintained in a mouse facility under controlled environmental conditions.

In addition to the parental strains and the F1 hybrids, we studied 35 BXD lines with an average of six mice per sex and line, resulting in a total of 581 mice. Liver fibrosis was induced at eight weeks of age. For a period of six weeks, CCl_4_ was administered by intraperitoneal injections twice weekly (0.7 mg CCl_4_/kg body weight in mineral oil, final volume 50 µl). Forty-eight hours after the last injection, the animals were anaesthetized with isofluran and killed by cervical dislocation. Instantly, blood was collected from the vena cava inferior, and tissue samples of liver and spleen were harvested. Liver samples were divided into five separate lobes. Whole liver weight, spleen and body weights were noted.

The animal studies were conducted according to all relevant welfare regulations and the Animal Care and Use Committee for Saarland University approved the protocols (TV Nr. 10/2008).

### Phenotypic characterization of hepatic fibrosis

We measured the following quantitative CCl_4_-induced phenotypes: hepatic collagen contents (hydroxyproline levels and collagen areas) as quantitative measures and fibrosis stage as semiquantitative measure in histological liver sections. In 35 BXD lines, C57BL/6J, DBA/2J and F1 hybrids, collagen contents were determined in liver hydrolysates from snap frozen specimens of the right hepatic lobe. The assay is based on photometric measurement of the collagen specific amino acid hydroxyproline (Hyp) and follows the slightly revised protocol of Jamall et al. [18], [22].

For the histological assessment of liver injury, formalin-fixed left lobes (4%, v/v) were available from 29 BXD lines, strains C57BL/6J and DBA/2J as well as B6D2 F1 hybrids. Each lobe was cut into 3–4 cross sections and embedded in one paraffin block. To detect collagen fibers, paraffin sections were stained with Sirius red [18]. The staging of fibrosis was performed using a semi-quantitative scoring system adapted from the system of Batts and Ludwig [18], [23], [24], principally differentiating the stages F0 to F4 (‘F-scores’).

Furthermore, stained collagen areas were quantified by morphometric analysis, using a semiautomatic system for image analysis (Stingray F146C IRF Medical camera, ½″ type progressive scan CCD, Germany, and HistoQuant image morphometry software, 3DHistech, Budapest, Ungary). Mean collagenous areas (µm^2^) were calculated by setting a threshold capturing Sirius red stained areas of collagen. One representative field (magnification 100x) was chosen from each liver section (avoiding arteries of a diameter >100 µm), and the mean percentage of the stained area to whole area (field of vision) was calculated. Liver injury was assessed by serum alanine aminotransferase (ALT) activity. After CCl_4_ challenge, blood was collected in a terminal procedure as described above. Blood was centrifuged for 20 min with 2000×*g* at 4°C. Serum was diluted with 0.9% (v/v) NaCl, and ALT levels were determined in the central laboratory of Saarland University Medical Center according to the IFCC reference method (Cobas, Roche Hitachi, Indianapolis, IN) [25].

### Microarray analysis of hepatic expression profiles

Total RNA was isolated from snap frozen individual liver samples (∼30 mg) of 30 BXD strains, the parental strains and B6D2 F1 hybrids, using the RNeasy mini kit (Qiagen, Hilden, Germany). Three female mice per strain were analyzed after CCl_4_ treatment for six weeks as described above, resulting in a total of 99 liver samples.

RNA quality was verified by measurement of the RNA integrity number (2100 Bioanalyzer, Agilent, Santa Clara, CA). Whole genome profiles of the fibrotic livers were performed using Gene Chip Mouse Gene 1.0 ST arrays (Affymetrix, Santa Clara, CA). For the normalization of robust multi-array average (RMA) intensity estimates of each transcript, RMAs were transformed into log_2_-values. Then the data of each single array was converted to their Z-scores, so that each array has a mean of 0 and a standard deviation of 1. Subsequently all values were multiplied by 2, and a value of 8 was added. Accordingly, all final values are positive and all datasets have an average of 8 units. One unit of expression on this scale resembles approximately a two-fold difference in expression.

The Affymetrix expression data set comprised a total of 34,760 records, which were assigned to 22,349 annotated genes. We determined the mean gene expression values for each probe set. As internal control, the strain distribution patterns of eQTLs with a Mendelian (monogenic) expression pattern (e.g. *Alad*, delta-aminolevulinate dehydratase; *Hc*, hemolytic complement; *Tceanc2*, transcription elongation factor A) were determined to show a perfect match to those of their closest markers, verifying that there were no errors of strain assignment in this data set (not shown).

All normalized transcript data are available in the GeneNetwork database (accession ID GN325, database name SUH BXD Liver CCl4-treated Affy Mouse Gene 1.0 ST (Jun11) RMA). GeneNetwork is an open-access database that collates genomic information of diverse experimental crosses and reference panels as well as phenotypic data from miscellaneous research groups [26].

### Statistics

Data generation, statistical analysis and graph creation were performed with SPSS Statistics 21 (IBM, Ehningen, Germany). As appropriate, mean and median values were further used for QTL analysis. Phenotypic robustness for each strain was assessed by the standard errors of the means. Mean and median values were trimmed by identifying and omitting outliers after graphical inspection of the data in box plots. Trait values of each BXD line were analyzed for each sex separately, as well as for the combined data sets of female and male mice. All fibrosis trait data were uploaded into the GeneNetwork database (accession IDs 14355–14396). Pearson's correlation was used to correlate fibrosis data among themselves and to BXD phenotypes.

Heritability (h^2^) of the fibrosis traits was calculated as the ratios of between-strain variances to within-strain variances in sex-specific data sets [27]. Within-strain and between-strain variances were calculated with analysis of variance (ANOVA) [28].

#### pQTL analysis

The identification and mapping of phenotypic QTLs (pQTLs) was performed by linking trait data to genotypes at known genetic marker loci [13], [29]. All phenotypic data were integrated into the GeneNetwork database. For the identification of single QTLs, interval mapping analyses were performed across all chromosomes [30]. The parental strains were included in all mapping analysis. For composite interval mapping (CIM), a single genetic marker representing an identified QTL region was included as covariate, increasing the power to identify QTLs on other chromosomes by removing the effect of the pre-eminent QTL [31]. CIM was performed for every phenotype, choosing genetic markers with highest likelihood ratio statistics (LRS) at each single QTL region or interacting loci from pairwise interaction scans.

The significance of a hypothetical QTL was estimated from the LRS [30]. Genome-wide significance was evaluated by testing 2,000 permutations [32], which specified a significant threshold corresponding to a genome-wide p-value (p_G_) of 0.05, and a suggestive threshold corresponding to p_G_ = 0.63. Confidence intervals of chromosomal regions spanning QTL positions were specified as 1.5 logarithm of the odds (LOD) support intervals.

#### eQTL analysis

In this study, we aimed to infer causal mechanisms for phenotypic variation. Observing effects on gene expression that result from variants in the identified genomic region increases the confidence that this locus harbors causal candidates underlying the phenotype. Therefore we followed a pre-defined selection strategy for candidate genes, herein we chose to restrict our candidate search to the genes located in significant and phenotype-overlapping *pQTL* regions (Figure S1 in File S1). Genes were identified using the GeneNetwork/UCSC Genome browser [33]. Since gene expression is, at least in part, heritable, differences in mRNA expression levels over the panel of BXD lines can be used to map regulatory expression quantitative trait loci (eQTLs). Therefore hepatic expression levels were used as quantitative traits and implemented into interval mapping analyses to identify regulatory loci. An expression quantitative trait locus (QTL) for a specific transcript was denoted *cis*QTL if the associated marker was localized within a 10 Mb distance of the gene position [34]. The respective gene was called a *cis*-regulated quantitative trait gene (*cis*QTG).

#### Integrated search for candidate genes

The lists of *cis*QTGs for the fibrosis phenotypes were refined following an explorative *in silico* data analysis (Figure S2 in File S1). We applied the following selection criteria, of which at least one had to be fulfilled by the *cis*QTG to qualify as a potential fibrogenic susceptibility gene: (i) significant Pearson's correlation coefficient (p<0.05 corresponding to r>0.36 or r<−0.36) between *cis*QTG and any of the fibrosis phenotypes (collagen area, Hyp, F-score); (ii) non-synonymous single nucleotide polymorphism (nsSNP) that differs between the parental strains B6 and D2. nsSNPs leading to amino acid substitutions in the coding regions were identified using the GeneNetwork variant browser; and (iii) differential hepatic regulation of *cis*QTGs in expression data sets after CCl_4_ challenge and saline treated control livers. QTL regions were matched using QTLminer [35] and the GeneNetwork dataset for saline treated livers in females (accession ID GN312, database name GenEx BXD Sal Liver Affy M430 2.0 (Feb11) RMA Females).

#### Gene network construction

Pairwise correlation estimates of the expression values of designated candidate genes were calculated as Pearson's correlation coefficient and presented in a circular network graph using Cytoscape software [36]. For purposes of illustration the intra-chromosomal correlations of genes on the same chromosome were omitted, and only inter-chromosomal correlations are shown in the network. Edge colors represent positive or negative correlations with r>0.36 or r<−0.36, and node sizes indicate the connectivity of the genes.

All candidate genes including the three fibrosis phenotypes were additionally illustrated in a QTL heatmap. This heatmap visualizes the p-values of regulatory loci identified by genome-wide linkage analysis of the traits, computed on the basis of permuation tests (n = 1,000).

### Data access

All trait data were uploaded into the GeneNetwork database “BXD Published Phenotypes Database” (http://www.genenetwork.org/): Fibrosis phenotype data (CCl_4_ treated livers) accession IDs 14355–14396; expression dataset (CCl_4_ treated livers, females) accession ID GN325, database name SUH BXD liver Affy Mouse Gene 1.0 ST (Jun11) RMA; expression dataset (saline treated livers, females) accession ID GN312, database name GenEx BXD Sal Liver Affy M430 2.0 (Feb11) RMA Females, provided by courtesy of Dr. Robert Rooney, Genome Explorations (Memphis, TN).

## Results

### Liver fibrosis in parental strains

**Figure 1:**
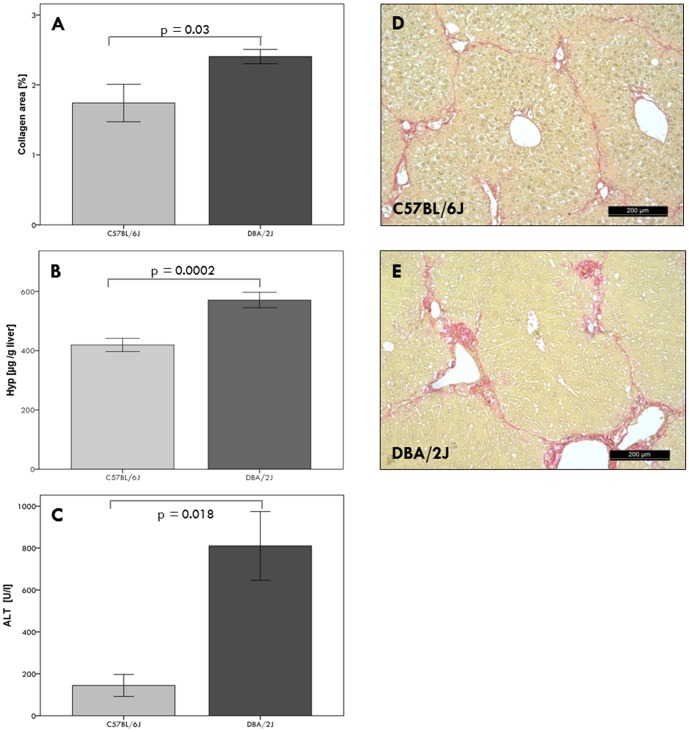
Phenotypic characterization of parental strains after six weeks of CCl_4_ injections. Liver fibrosis was assessed by morphometric (A) and biochemical (B) measurement of hepatic collagen (Hyp) contents. Hepatic inflammation was measured by serum ALT activities (C). Sirius red staining of hepatic collagen showed circumferential fibrosis in C57BL/6J mice (D) and pronounced fibrosis in DBA/2J mice (E), corresponding to mean F-scores of 2.0±0.1 and 3.9±0.1, respectively.

A significant (p<0.0001) accumulation of collagen was observed in all livers after six weeks of CCl_4_ challenge in comparison to untreated mice. The mortality rate of mice after CCl_4_ challenge was 8%. The parental inbred strain DBA/2J is more susceptible to liver fibrosis than the C57BL/6J strain, indicated herein by significantly (p<0.05) increased collagen areas and higher hepatic collagen (Hyp) contents; this is paralleled by liver injury as assessed by serum ALT activities (Figure 1).

### Liver fibrosis phenotypes in the BXD reference panel

**Figure 2:**
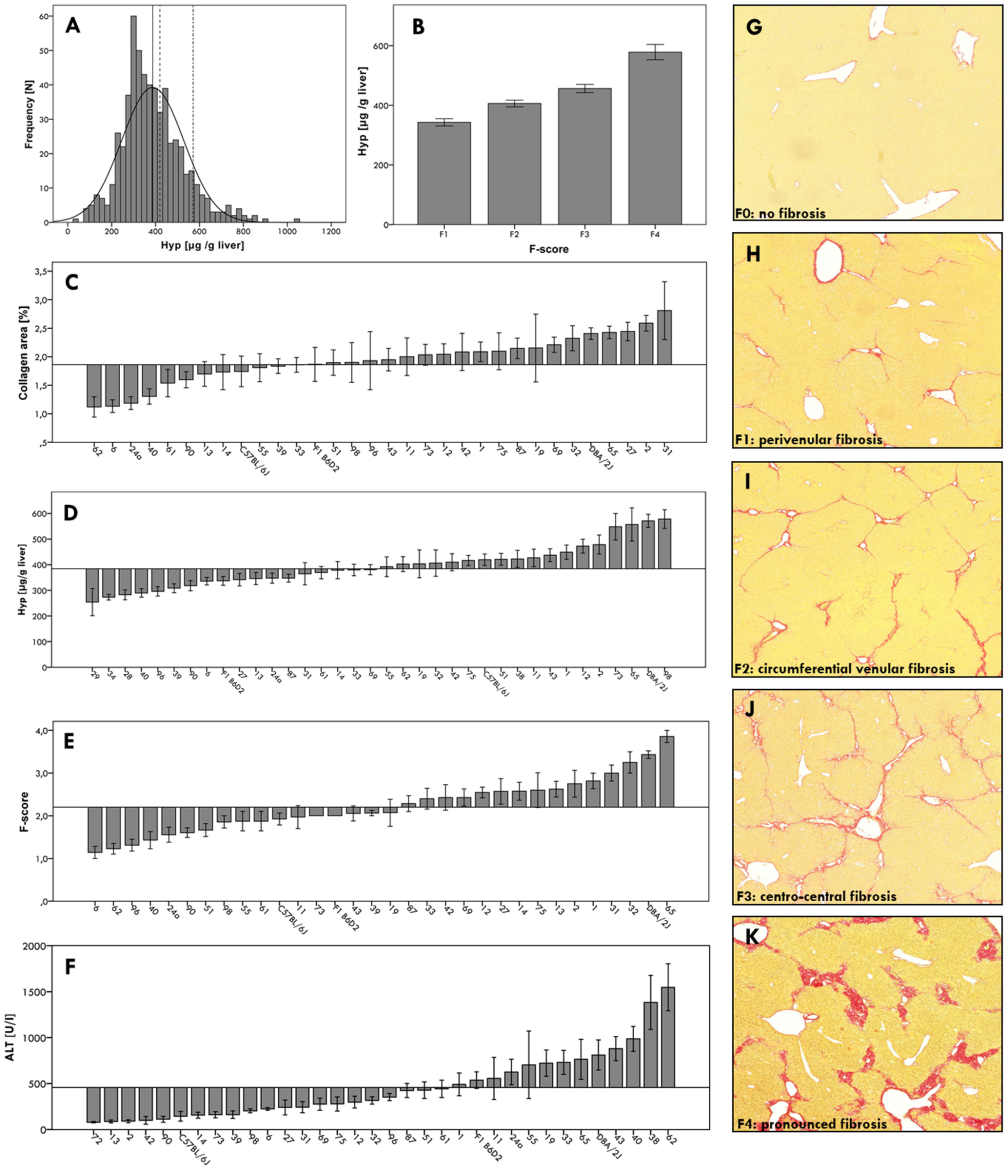
Phenotypic characterization of the BXD reference panel after six weeks of CCl_4_ injections. (A) Histogram illustrating the distribution of hepatic collagen contents in BXD mice. The mean hepatic collagen concentrations (± SE)are indicated by vertical lines (solid line: BXD recombinant inbred lines, 386.9±5.9 µg Hyp/g liver; dashed line: C57BL/6J (B6) inbred strain, 419.3±22.4 µg/g; dash-dot line: DBA/2J (D2) inbred strain, 570.9±25.9 µg/g). (B) Mean hepatic collagen contents (± SE) stratified according to F-score categories (F1–F4). (C–E) Strain specific mean (± SE) phenotype values compared to the overall means of all mice phenotyped (29–35 BXD lines, strains C57BL/6J and DBA/2J, B6D2 F1 hybrids), which are represented by the horizontal lines. (C) Hepatic collagen areas (mean ± SE: 2.4±0.1%); (D) hepatic collagen (Hyp) contents (386.9±5.9 µg/g); (E) fibrosis stages (F-scores, 2.3±0.1); (F) serum ALT activities (419.4±25.3 U/l). (G)–(K) Representative liver sections of diverse BXD strains after CCl_4_ treatment, illustrating increasing F-scores from F0 to F4. (G) F0: no fibrosis; (H) F1: perivenular fibrosis, initially forming collagen bridges; (I) F2: circumferential venular fibrosis with incomplete bridging; (J) F3: centro-central fibrosis with complete bridging; (K) F4: pronounced fibrosis with complete and broadened collagen bridges.

We noted marked differences in liver fibrosis among the BXD lines. Hepatic collagen contents varied widely (mean ± SD 386.9±141.5 µg Hyp/g liver; Figure 2A), and histopathological fibrosis scores correlated significantly (p<0.001) with hepatic collagen levels (Figure 2B). Furthermore, we observed significant (p<0.0001) line differences with respect to hepatic collagen levels and fibrosis scores (Figures 2C–E) as well as clinical-chemical parameters (ALT) of liver damage (Figure 2F). Semiquantitative fibrosis scores ranged from F1 (perivenular fibrosis) to F4 (pronounced fibrosis) (Figure 2G–K).

Heritability of liver fibrosis as determined by mean h^2^ values was similar for all fibrosis traits, ranging from 0.51±0.18 (Hyp) to 0.57±0.02 (F-score) and 0.59±0.01 (collagen area). Overall, hepatic collagen area was identified as the most heritable trait (h^2^ = 0.59). h^2^ for hepatic Hyp concentrations showed a difference between male (0.36) and female mice (0.87), whereas h^2^ for the other traits did not differ between sexes.

### Genome-wide mapping of liver fibrosis phenotypes (pQTLs)

Single QTL genome scans identified 28 trait associated loci (Table S1 in File S2) with LRS scores above the suggestive threshold (see *Methods*) affecting liver fibrosis phenotypes. Among these significantly linked loci were detected by composite interval mapping (p_G_<0.05) on chromosomes 4, 5, 7, 12, and 17 (Table 1, Figure S3 in File S1). For all loci except the QTL on chromosome 12, alleles of the fibrosis-susceptible strain DBA/2J increased the trait values (Table S1 in File S2). Five QTLs on chromosomes 2, 5, 7, 13 and 15 conferred susceptibility to more than one phenotype (Table S2 in File S2), whereas the loci on chromosomes 4, 12 and 17 were specific for a single phenotype.

**Table 1:** Chromosomal regions of pQTLs with significant genome-wide LRS values determined by single QTL scans and CIM.

Phenotype	pQTL (Chr)	LRS (max)	SNP (max)	1.5 LOD support interval (Mb)	Additive allele effect (−) C57BL/6J (+) DBA/2J	Dataset
Collagen area	5	18.1	mCV23582150 - rs6392739	3.1–20.1	0.323	male/both
Collagen area	5	23.1	rs3678577 - rs6167407	85.1–97.9	0.451	female/both
Hyp	4	17.4	rs6254381 - rs13477745	55.1–73.9	58.957	female/both
Hyp	7	16.3	rs3703247 - rs8255275	52.8–56.7	56.761	female/both
Hyp	12	25.0	rs3716547 - rs13481511	60.5–73.3	−77.257	female/both
F-score	7	20.3	rs3703247 - rs8255275	48.2–53.7	0.562	male/both
F-score	17	22.0	rs13483077 - rs13483081	64.9–71.1	0.516	female

Abbreviations and definitions: **pQTL (chr):** chromosomal position of quantitative trait locus; **LRS (max):** likelihood ratio statistic, maximum association between genotype and phenotype variation; **SNP (max):** single nucleotide polymorphism with maximum LRS in QTL region; **1.5 LOD support interval (Mb):** chromosomal region in Megabases spanning QTL position; **Additive allele effect**: estimate of a change in the average phenotype by substitution of one parental allele by another at a given marker position; **(−)** values indicate an increase of phenotype by C57BL/6J allele, **(+)** values an increase of phenotype by DBA/2J allele; **Dataset:** dataset in which the QTL was identified; **Hyp**: hydroxyproline; CIM: composite interval mapping.

We found that 16 of 28 QTLs (46%) were sex-specific, i.e. they were only found in data from male or female mice (Table S1 in File S2). The remaining 11 loci were detected in the combined datasets; but showed significant effects either in male or female, consistent with a predominant phenotypic effect of one sex. In addition, the chromosome 2 QTL at 174.5–181.5 Mb was detected in all datasets tested. QTLs for hepatic Hyp levels were mainly based on female datasets; this was in line with the higher h^2^ scores for this trait in female mice. For collagen area and F-score QTLs, no predominance of a single sex was observed.

### Genome-wide mapping of fibrosis-associated eQTLs

**Figure 3:**
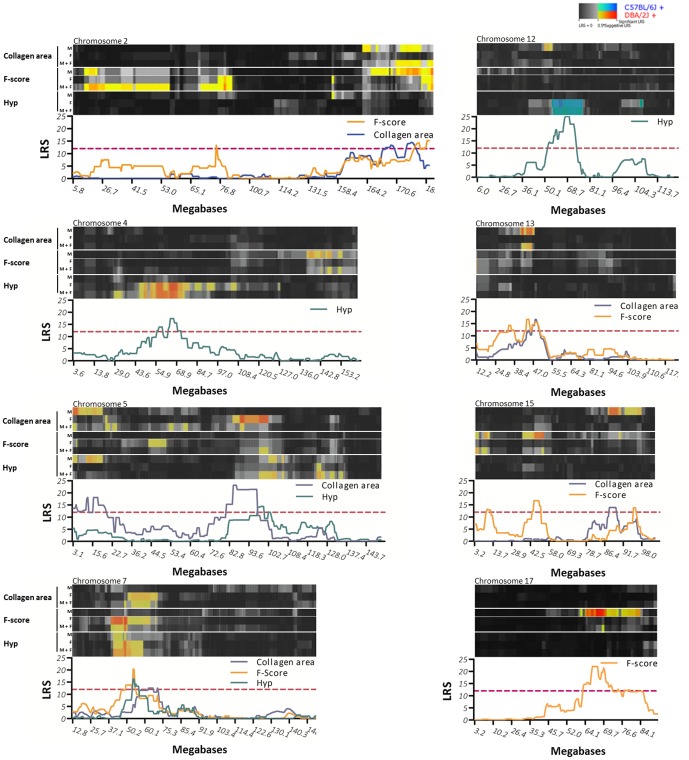
QTLs for hepatic fibrosis in the BXD murine reference population. The heatmaps represent significant interval mapping results on the indicated mouse chromosomes, separately for male and female mice as well as the combined data set (without inclusion of covariates); the QTL plots below illustrate composite interval mapping results (with ‘background’ QTLs as covariates, restricted to significant QTLs or overlapping loci for different phenotypes). Color coding of the heatmaps is as follows: Grey/black regions indicate the absence of genotype to phenotype linkage. Blue to green regions correspond to suggestive and significant linkage, respectively, with C57BL/6J alleles being associated with higher trait values. Red to yellow regions correspond to suggestive and significant linkage, respectively, with an association of DBA/2J alleles with higher values.

Nine pQTL regions on chromosomes 2, 4, 5, 7, 12, 13, 15 and 17 were further dissected using eQTL mapping (Figure 3). The nine selected loci either conferred significant linkage or were associated to more than one phenotype. eQTLs were *cis*-regulatory loci (*cis*QTLs) or *trans*-regulatory loci (*trans*QTLs) (see *Methods*). By mapping *cis*-regulatory eQTLs within the nine most significant pQTL regions, we identified fibrosis-associated expression patterns that were locally regulated within the pQTLs. Table 1 summarizes the results of the eQTL analysis: On average, the pQTLs spanned an interval of 15.2 Mb, and these pQTL regions contained a total number of 1,351 annotated genes. The highest LRS score was observed for the QTL on chromosome 12, the only locus for which fibrosis susceptibility was conferred by the C57BL/6J allele. The QTL on chromosome 7 was the largest QTL with a high gene density. Overall, we identified 68 regulatory markers (eQTLs) within the pQTL regions. Thirty regulatory markers were identified as *cis*QTLs. Using the upper limit of suggestive thresholds for genome-wide significance as determined by permutation tests (LRS≥12.0), these markers were linked to 85 genes in close proximity (<10 Mb). The associated genes are potentially *cis*-regulated genes (*cis*QTGs) in the pQTL regions. Of note, all *cis*QTLs also demonstrate *trans*-regulation of additional genes outside the pQTL regions (not shown). Applying this combined analysis of pQTLs and eQTLs, we reduced the number of potential fibrosis candidate genes from 1,351 to 85 (Table 2, Figure S2 in File S1).

**Table 2:** Summary of fibrosis-associated pQTL and eQTL regions.

pQTL (Chr)	pQTL position (Mb)	Phenotype	LRS (max)	Size of pQTL region (Mb)	Genes in pQTL region	eQTLs in pQTL region	*cis*QTLs in pQTL region	*cis*QTGs in pQTL region LRS≥12.0
2	167.7–181.5	Collagen area F-score	15.2	13.8	170	7	2	4
4	55.1–73.9	Hyp	17.4	17.9	123	6	6	13
5	3.1–20.1	Collagen area	18.1	17.0	103	8	1	1
5	82.8–103.9	Collagen area Hyp	23.1	21.1	182	10	5	12
7	48.2–74.2	Collagen area Hyp F-score	20.3	26.0	396	9	7	34
12	60.5–73.3	Hyp	25.0	12.8	90	2	1	5
13	44.2–52.7	Collagen area F-score	16.7	8.5	81	4	2	2
15	82.3–95.9	Collagen area F-score	13.9	13.6	154	11	6	17
17	64.9–71.1	F-score	22.0	6.2	52	5	0	0
**Total**				**15.2**	**1,351**	**68**	**30**	**85**

Abbreviations and definitions: **pQTL (chr):** position of phenotypic (p) QTL; **pQTL Position (Mb)**: chromosomal position in Megabases; **LRS (max):** likelihood ratio statistic, maximum association detected in pQTL analysis; **Size of pQTL region (Mb):** size of 1.5 LOD support interval of the QTL; **Genes in pQTL region**: all genes localized in a pQTL region; **eQTLs in pQTL region**: regulatory genetic markers in pQTL region; ***cis*** **QTLs in pQTL region**: genetic markers in the pQTL region, regulating genes within a 10 Mb distance; ***cis*** **QTGs in pQTL region**: genes in the pQTL region (regulated by markers within a 10 Mb distance) with LRS≥12.0.

### Selecting fibrogenic candidate genes

In further selection steps, we inferred key regulatory candidates of fibrosis among the 85 *cis*QTGs. This strategy refined the list of 85 *cis*QTGs to 55 potential profibrogenic candidate genes that fulfilled at least one selection criterion (Table S3 in File S2). First, Pearson's correlation of *cis*QTG expression with any fibrosis phenotype (collagen area, Hyp, F-score) identified 30 significantly (p<0.05) correlated genes (indicated by dark gray boxes). Next, we determined covariance of hepatic mRNA expression patterns in unchallenged and CCl_4_ challenged livers of the BXD reference lines, using QTLminer analysis. This analysis indicated that eQTLs could be distinguished into (A) fibrosis-specific *cis*QTLs that showed differential regulation between the basal state and after fibrosis induction, and (B) fibrosis-independent *cis*QTLs, i.e. the genes were *cis-*regulated in both groups. We speculate that differential regulation of class A genes in fibrotic livers identifies more relevant modifiers of fibrogenesis. In total, 45 *cis*QTGs were differentially regulated (class A), while 40 *cis*QTGs were *cis*-regulated in both normal and fibrotic livers (class B). Finally, we identified 169 genes in the pQTL regions with nsSNPs that segregated between the two parental lines C57BL/6J and DBA/2J. Among the 85 *cis*QTGs, 22 have nsSNPs in coding regions.

In summary, 55 candidate genes listed in Table S3 in File S2 fulfilled at least one criterion and were either significantly correlated to a specific fibrosis phenotype, differentially regulated, or contained an amino acid substitution. Only 31 genes passed at least two criteria, and merely eleven genes fulfilled all three criteria: *Afm* (afamin), *Fan1 (FANCD2/FANCI-associated nuclease 1)*, *Hsd17b14 (hydroxysteroid (17-beta) dehydrogenase 14)*, *Napsa (napsin A aspartic peptidase)*, *Nomo (nodal modulator 1)*, *Nin* (ninein), *Susd1 (sushi domain containing 1)*, and four members of the kallikrein 1b family (5, 21, 22 and 26). Most candidate genes are located on chromosome 7 (n = 21), followed by chromosomes 5 (n = 11), 15 (n = 9), 4 (n = 7), 12 (n = 4), 2 (n = 2), and 13 (n = 1). The QTLs on chromosome 5 (3.1–20.1 Mb) and chromosome 17 contained no *cis*QTG complying with the criteria.

### Generating a fibrosis gene network

**Figure 4:**
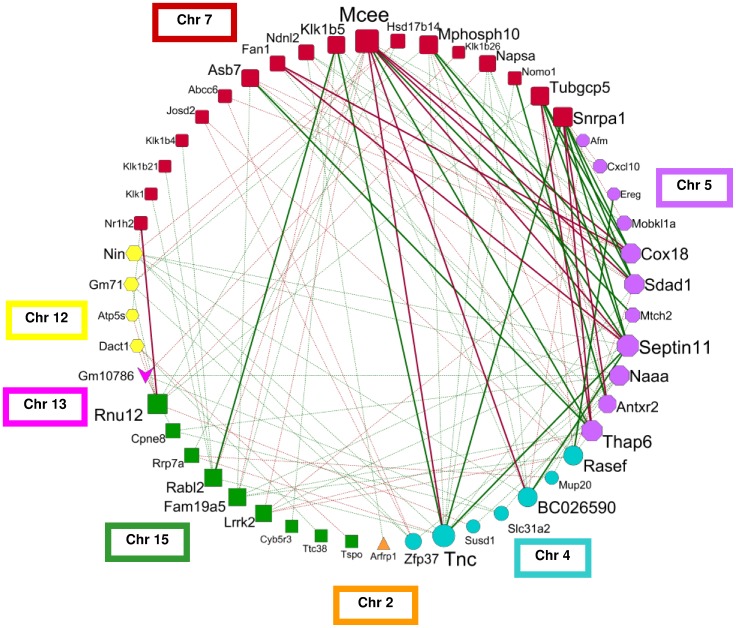
Fibrosis network graph generated by correlating the hepatic expression levels of the candidate genes. The graph presents the inter-chromosomal correlations of 51 genes, except for the four candidate genes *Adamts17*, *Gm9860*, *Klk22 and Ogfr* with exclusively intra-chromosomal correlations. Node color and shape illustrate the chromosomal localization of the gene. The size of each node indicates the degree of connectivity, with larger nodes having higher number of correlated genes. The edges show Pearson's correlation coefficients (r) as follows: solid green lines: r>0.5; solid red lines: r<−0.5; dotted green lines r>0.36; dotted red line r<−0.36.

We generated a circular network graph (Figure 4), using the pairwise correlation estimates of hepatic expression levels for the 55 fibrosis candidate genes (Table S3 in File S2). The nodes represent the genes, which were arranged according to their chromosomal localizations, and edges show significant (p<0.05) inter-chromosomal correlations between gene expression levels. This unique network contains a total of 115 inter-chromosomal correlations; for reasons of simplicity the graph does not show the 93 intra-chromosomal correlations. The genes with the highest inter-chromosomal connectivity are *Mcee* (n = 13), *Tnc* (n = 12), *Sept11* (n = 11), *and Thap6* (n = 10). Additional analyses of our transcriptomic dataset showed that *Tnc* highly correlates with other fibrosis-associated genes, in particular with hepatic expression levels of collagens (*Col1a2*: r = 0.76; p = 0.002; *Col3a1*: r = 0.75; p = 0.001) and transforming growth factor β 1 (*Tgfb1*: r = 0.74; p = 0.002).

In addition, we illustrated the regulatory eQTLs of the candidate genes and the loci for fibrosis phenotypes (pQTL) in a QTL heatmap (Figure S4 in File S1). Regulatory gene clusters on chromosomes 2, 4, 5, 7 and 12 co-localized with loci of fibrosis phenotypes, as indicated by arrows in the QTL heatmap. The large eQTL on chromosome 7 showed two differentially regulated gene clusters: The genes located on distal chromosome 7 (63.1–74.2 Mb) displayed additional regulatory loci on chromosome 5, which were absent for the genes between 51.2 and 53.5 Mb.

## Discussion

Previously we identified individual loci that confer genetic susceptibility to hepatic fibrosis in different experimental crosses of inbred mice [6], [7], [18]. Here we report the first systems genetics analysis of fibrosis in the BXD murine reference panel that allows the integration of multiple traits [13]. The genetically mosaic BXD inbred lines display significant variation of quantitative fibrosis phenotypes, consistent with polygenic inheritance of liver fibrosis [1], [4], [5]. By correlating phenotypes and known BXD genotypes in a genome-wide QTL analysis, we identified multiple pQTLs, nine with genome-wide significance and several with sex-dependent effects. Sex-specific differences are observed in various liver diseases such as (non-)alcoholic fatty liver diseases and hemochromatosis and might be due to sex hormone-regulated mechanisms or sex-specific gene variants. In addition to the phenotypic characterization of fibrosis, we generated a comprehensive expression dataset that represents the first genome-wide transcriptome analysis of hepatic fibrosis in a murine reference panel.

By stepwise bioinformatic analyses [37], we were able to reduce the number of 1,351 genes located in the nine pQTL regions to a set of 55 creedal fibrogenic candidate genes (Figure S2 in File S1). For this analysis, we focused our search on the genes that are *cis*-regulated during fibrogenesis. To minimize the false discovery rate, the genes underwent a subsequent careful explorative analysis. They were considered to be relevant for fibrosis when their expression levels correlated with the fibrosis traits or showed differential regulation in healthy and fibrotic livers. In addition, we screened these *cis*QTGs for nsSNPs in the parental strains of the BXD panel, which might structurally and/or functionally affect protein functions. After all these steps, eleven genes fulfilled all selection criteria (*Afm, Fan1, Hsd17b14, Klk1b5, Klk1b21, Klk1b22, Klk1b26, Napsa, Nomo, Nin, Susd1*). For the majority of these genes there is no established connection to hepatic fibrosis or little information about their function in liver, although kallikreins exert known functions in the activation of inflammation, wound healing, and liver regeneration [38]. In particular, we consider *Afm* as interesting candidate with a so far unknown role in hepatic fibrogenesis. *Afm* is a member of the albumin gene family that was shown to be differentially regulated by hepatocyte nuclear factors 1α and 1β in mice [39]. Interestingly it functions as carrier of vitamin E [40], which has recently been reported to ameliorate liver fibrosis in fatty liver disease in mice and humans [41], [42]. A study by Kim et al. [43] revealed that afamin acts as a chemokine activating the Akt-signalling cascade, at least in osteoblasts. Because expression profiling showed *Afm* to be differentially expressed in mouse liver, this observation suggests similar regulatory effects across organs.

A further proof of principle is that several of the significant QTL regions include potential candidates that have previously been associated with fibrosis progression, in particular the chemokine ligand *Cxcl10, Nr1h2* (nuclear receptor subfamily 1, group H, member 2, a.k.a. LXR), and *Tnc* (tenascin C). *Cxcl10* encodes a chemokine that promotes hepatic inflammation by leukocyte recruitment [44]. Additional *in vitro* experiments in primary mouse hepatocytes detected a time-dependent induction of *Cxcl10* expression levels after treatment with *Tgfb1*, supporting its role as profibrogenic candidate gene (R.H., R.L. and F.L.; unpublished observations). The nuclear receptor LXR might exert antifibrotic effects, since it was shown to reduce hepatic stellate cell activation, and therefore inhibit the production of profibrogenic cytokines [45]. *Tnc* is an extracellular matrix glycoprotein expressed by hepatic stellate cells and myofibroblasts in liver, where it increases cytokine expression and ameliorates leukocyte transmigration [46], [47]. Whereas *Tnc* expression is absent in naïve livers, it is strongly induced during enhanced cell turnover as seen in wound repair [48], [49]. It also represents an endogenous ligand of *Tlr4*, which promotes innate immune responses during fibrogenesis [50], [51]. Further correlation network analysis of our transcriptome datasets showed that *Tnc* is highly interconnected with other fibrosis-associated genes (R.H. and F.L., unpublished observations); in particular, it is significantly correlated with the hepatic expression levels of collagens (*Col1a2*: r = 0.76; p = 0.002; *Col3a1*: r = 0.75; p = 0.001) and *Tgfb1* (r = 0.74; p = 0.002).

To illustrate the complexity of fibrosis susceptibility, we combined the genes and their expression correlations in a large ‘fibrosis network’, indicating the inter-chromosomal connectivity of fibrogenic genes. In addition, the genetic architecture of co-regulated gene networks were visualized by eQTL heatmaps, which show that the regulatory gene clusters of fibrosis susceptibility genes co-localize with pQTLs (Figure S4 in File S1). These findings reflect the selection concept for the candidate genes, with gene clusters representing networks with a causal relationship to fibrosis phenotypes. Gene repression studies (e.g. siRNA) might help to further dissect the directionality of the gene effects and fibrosis gene networks.

Recently genome-wide association studies (GWAS) in patients with chronic liver diseases have identified profibrogenic gene variants [5]. Genes associated with fibrosis progression in patients with chronic HCV infection were *MERTK* (c-mer proto-oncogene tyrosine kinase), *RNF7* (ring finger protein 7), and *TULP1* (tubby like protein 1) [12]. In contrast, the genes *PNPLA3* (adiponutrin), *GCKR* (glucokinase regulator) and *TRIB1* (tribbles homolog 1) were associated with fibrosis phenotypes in non-alcoholic fatty liver disease [52], with *PNPLA3* demonstrating the most consistent effects across studies [53]. Although our expression dataset shows that all murine orthologs are expressed in fibrotic mouse liver (mean expression scores 7.2–10.4; R.H. and F.L., unpublished observations), solely *Pnpla3* was located in a pQTL on chromosome 15. However, it was apparently not *cis*-regulated in our panel and did not display nsSNPs in the parental strains. The lack of cross-species validation might be due to the distinct induction of fibrosis in our model, the specific charcteristics of the patients included in the GWAS (viral hepatitis, fatty liver disease), different phenotypic effect sizes across species, or non-polymorphic regions within the BXD panel.

This study illustrates how advances in the methodologies of systems genetics with the use of a murine reference panel lead to the identification of a potential disease network for liver fibrosis. The BXD lines represent an appropriate reference population with phenotypic segregation of fibrosis phenotypes due to genetic variation. Our findings indicate that it is essential not to focus on single fibrogenic QTLs, but on gene clusters as modifiers of fibrosis susceptibility. Although several existing confounders are being controlled for within this approach, further developments in gene mapping and functional validation will contribute to the translation of our experimental findings to patients with liver fibrosis.

## Supporting Information

File S1 Figure S1, Graphical overview of the experimental setup for the integrative analysis of pQTLs and eQTLs in the BXD murine reference panel. Abbreviations: BXD, recombinant inbred lines based on parental strains C57BL/6J and DBA/2J; CCl_4_, carbon tetrachloride; DNA, deoxyribonucleic acid; eQTL, expression quantitative trait locus; F-score, fibrosis score; Hyp, hydroxyproline; n, number; pQTL, phenotypic quantitative trait locus. Figure S2, Study design and strategy for the selection of candidate genes. Genome-wide association studies of CCl_4_ treated BXD lines identified phenotype-associated QTLs (pQTLs). The 1,351 genes located in significant pQTLs were investigated further by eQTL analyses (see *Methods*). This allowed the differentiation of local (*cis-*) or distant (*trans*)-regulation of gene expression. *Cis*-regulated genes (*cis*QTGs) underwent the following three selection steps to refine the list of candidate genes: I) *cis*QTGs with significant correlation with fibrosis phenotypes; II) fibrosis-specific *cis*QTGs that show differential regulation between basal state and after the induction of fibrosis; and III) *cis*QTGs with non-synonymous (ns) SNPs segregating in strains C57BL/6J and DBA/2J. *cis*QTGs complying with one of the three criteria were considered as creedal candidate genes. In total, 55 candidate genes were included into the fibrosis network. Abbreviations: *cis*QTGs, *cis*-regulated genes; eQTL, expression quantitative trait locus; F-score, fibrosis score; Hyp, hydroxyproline; n, number; nsSNP non-synonymous single nucleotide polymorphism; pQTL, phenotypic quantitative trait locus; P_G_, genome-wide p-value. Figure S3, Single QTL scans identifying significant loci for each fibrosis phenotype. Legend on top left: empirical genome-wide significance thresholds of LRS values, significant (pink line), suggestive (grey line); additive allele effect, DBA/2J alleles (green), C57BL/6J alleles (red). Traits: (A–B) collagen area, (C–E) hepatic collagen (Hyp) concentration and (F-G) F-score. Figure S4, QTL heatmap of candidate genes and phenotypic data. The upper part lists the 55 candidate genes and the three phenotypes collagen area, F-score and Hyp; the lower part provides a QTL heatmap for all genes on the respective mouse chromosomes. Each vertical column illustrates a genome-wide eQTL analysis for the transcript levels on the BXD genome, represented as color-coded p-values. Color coding of the heat map is as follows: Grey/black regions indicate the abscence of genotype to phenotype linkage. Blue to green regions correspond to suggestive and significant linkage, respectively, with C57BL/6J alleles being associated with higher trait values. Red to yellow regions correspond to suggestive and significant linkage, respectively, with an association of DBA/2J alleles with higher values. Orange triangles indicate the localizations of the *cis*QTGs; the arrows point to their co-localizations with pQTLs.
(PDF)

File S2 Table S1, Chromosomal regions of pQTLs determined by single QTL scans and CIM. Table S2, Overlapping pQTL regions for different fibrosis phenotypes determined by single QTL scans and CIM. Table S3, Candidate genes of hepatic fibrogenesis.
(PDF)

## References

[1] Bataller R, Brenner DA (2005) Liver fibrosis. J Clin Invest 115: 209–218. 15690074 10.1172/JCI24282 PMC546435

[2] Hernandez-Gea V, Friedman SL (2011) Pathogenesis of liver fibrosis. Annu Rev Pathol 6: 425–456. 21073339 10.1146/annurev-pathol-011110-130246

[3] Poynard T, Bedossa P, Opolon P (1997) Natural history of liver fibrosis progression in patients with chronic hepatitis C. Lancet 349: 825–832. 9121257 10.1016/s0140-6736(96)07642-8

[4] Weber S, Gressner OA, Hall R, Grünhage F, Lammert F (2008) Genetic determinants in hepatic fibrosis: from experimental models to fibrogenic gene signatures in humans. Clin Liver Dis 12: 747–757. 18984464 10.1016/j.cld.2008.07.012

[5] Krawczyk M, Müllenbach R, Weber SN, Zimmer V, Lammert F (2010) Genome-wide association studies and genetic risk assessment of liver diseases. Nat Rev Gastroenterol Hepatol 7: 669–681. 21045792 10.1038/nrgastro.2010.170

[6] Hillebrandt S, Wasmuth HE, Weiskirchen R, Hellerbrand C, Keppeler H, et al (2005) Complement factor 5 is a quantitative trait gene that modifies liver fibrogenesis in mice and humans. Nat Genet 37: 835–843. 15995705 10.1038/ng1599

[7] Wasmuth HE, Lammert F, Zaldivar MM, Weiskirchen R, Hellerbrand C, et al (2009) Antifibrotic effects of CXCL9 and its receptor CXCR3 in livers of mice and humans. Gastroenterology 137: 309–319. 19344719 10.1053/j.gastro.2009.03.053 PMC2892869

[8] Wasmuth HE, Zaldivar MM, Berres ML, Werth A, Scholten D, et al (2008) The fractalkine receptor CX3CR1 is involved in liver fibrosis due to chronic hepatitis C infection. J Hepatol 48: 208–215. 18078680 10.1016/j.jhep.2007.09.008

[9] Krawczyk M, Grünhage F, Zimmer V, Lammert F (2011) Variant adiponutrin (PNPLA3) represents a common fibrosis risk gene: non-invasive elastography-based study in chronic liver disease. J Hepatol 55: 299–306. 21168459 10.1016/j.jhep.2010.10.042

[10] Pingitore P, Pirazzi C, Mancina RM, Motta BM, Indiveri C, et al.. (2013) Recombinant PNPLA3 protein shows triglyceride hydrolase activity and its I148M mutation results in loss of function. Biochim Biophys Acta: Epub ahead of print. 10.1016/j.bbalip.2013.12.006 24369119

[11] Speliotes EK, Yerges-Armstrong LM, Wu J, Hernaez R, Kim LJ, et al (2011) Genome-wide association analysis identifies variants associated with nonalcoholic fatty liver disease that have distinct effects on metabolic traits. PLoS Genet 7: e1001324. 21423719 10.1371/journal.pgen.1001324 PMC3053321

[12] Patin E, Kutalik Z, Guergnon J, Bibert S, Nalpas B, et al (2012) Genome-wide association study identifies variants associated with progression of liver fibrosis from HCV infection. Gastroenterology 143: 1244–1252. 22841784 10.1053/j.gastro.2012.07.097 PMC3756935

[13] Andreux PA, Williams EG, Koutnikova H, Houtkooper RH, Champy MF, et al (2012) Systems genetics of metabolism: the use of the BXD murine reference panel for multiscalar integration of traits. Cell 150: 1287–1299. 22939713 10.1016/j.cell.2012.08.012 PMC3604687

[14] Sieberts SK, Schadt EE (2007) Moving toward a system genetics view of disease. Mamm Genome 18: 389–401. 17653589 10.1007/s00335-007-9040-6 PMC1998874

[15] Peirce JL, Lu L, Gu J, Silver LM, Williams RW (2004) A new set of BXD recombinant inbred lines from advanced intercross populations in mice. BMC Genet 5: 7. 15117419 10.1186/1471-2156-5-7 PMC420238

[16] Taylor BA, Heiniger HJ, Meier H (1973) Genetic analysis of resistance to cadmium-induced testicular damage in mice. Proc Soc Exp Biol Med 143: 629–633. 4719448 10.3181/00379727-143-37380

[17] Gatti D, Maki A, Chesler EJ, Kirova R, Kosyk O, et al (2007) Genome-level analysis of genetic regulation of liver gene expression networks. Hepatology 46: 548–557. 17542012 10.1002/hep.21682 PMC3518845

[18] Hillebrandt S, Goos C, Matern S, Lammert F (2002) Genome-wide analysis of hepatic fibrosis in inbred mice identifies the susceptibility locus Hfib1 on chromosome 15. Gastroenterology 123: 2041–2051. 12454860 10.1053/gast.2002.37069

[19] Roberts A, Pardo-Manuel de Villena F, Wang W, McMillan L, Threadgill DW (2007) The polymorphism architecture of mouse genetic resources elucidated using genome-wide resequencing data: implications for QTL discovery and systems genetics. Mamm Genome 18: 473–481. 17674098 10.1007/s00335-007-9045-1 PMC1998888

[20] Williams RW (2006) Animal models in biomedical research: ethics, challenges, and opportunities. In: Principles of molecular medicine 2nd ed. pp. 53–60.

[21] Chesler EJ, Lu L, Shou S, Qu Y, Gu J, et al (2005) Complex trait analysis of gene expression uncovers polygenic and pleiotropic networks that modulate nervous system function. Nat Genet 37: 233–242. 15711545 10.1038/ng1518

[22] Jamall IS, Finelli VN, Que Hee SS (1981) A simple method to determine nanogram levels of 4-hydroxyproline in biological tissues. Anal Biochem 112: 70–75. 7258630 10.1016/0003-2697(81)90261-x

[23] Batts KP, Ludwig J (1995) Chronic hepatitis. An update on terminology and reporting. Am J Surg Pathol 19: 1409–1417. 7503362 10.1097/00000478-199512000-00007

[24] Ishak KG, Zimmerman HJ (1995) Morphologic spectrum of drug-induced hepatic disease. Gastroenterol Clin North Am 24: 759–786. 8749898

[25] Bergmeyer HU, Horder M, Rej R (1986) International Federation of Clinical Chemistry (IFCC) Scientific Committee, Analytical Section: approved recommendation (1985) on IFCC methods for the measurement of catalytic concentration of enzymes. Part 3. IFCC method for alanine aminotransferase (L-alanine: 2-oxoglutarate aminotransferase, EC 2.6.1.2). J Clin Chem Clin Biochem 24: 481–495. 3734711

[26] Wang J, Williams RW, Manly KF (2003) WebQTL: web-based complex trait analysis. Neuroinformatics 1: 299–308. 15043217 10.1385/NI:1:4:299

[27] Mhyre TR, Chesler EJ, Thiruchelvam M, Lungu C, Cory-Slechta DA, et al (2005) Heritability, correlations and in silico mapping of locomotor behavior and neurochemistry in inbred strains of mice. Genes Brain Behav 4: 209–228. 15924554 10.1111/j.1601-183X.2004.00102.x

[28] Visscher PM, Hill WG, Wray NR (2008) Heritability in the genomics era–concepts and misconceptions. Nat Rev Genet 9: 255–266. 18319743 10.1038/nrg2322

[29] Broman KW (2001) Review of statistical methods for QTL mapping in experimental crosses. Lab Anim 30: 44–52. 11469113

[30] Haley CS, Knott SA (1992) A simple regression method for mapping quantitative trait loci in line crosses using flanking markers. Heredity 69: 315–324. 16718932 10.1038/hdy.1992.131

[31] Lammert F, Wang DQ, Wittenburg H, Bouchard G, Hillebrandt S, et al (2002) Lith genes control mucin accumulation, cholesterol crystallization, and gallstone formation in A/J and AKR/J inbred mice. Hepatology 36: 1145–1154. 12395324 10.1053/jhep.2002.36821

[32] Churchill GA, Doerge RW (1994) Empirical threshold values for quantitative trait mapping. Genetics 138: 963–971. 7851788 10.1093/genetics/138.3.963 PMC1206241

[33] Kent WJ, Sugnet CW, Furey TS, Roskin KM, Pringle TH, et al (2002) The human genome browser at UCSC. Genome Res 12: 996–1006. 12045153 10.1101/gr.229102 PMC186604

[34] Brem RB, Yvert G, Clinton R, Kruglyak L (2002) Genetic dissection of transcriptional regulation in budding yeast. Science 296: 752–755. 11923494 10.1126/science.1069516

[35] Alberts R, Schughart K (2010) QTLminer: identifying genes regulating quantitative traits. BMC Bioinformatics 11: 516. 20950438 10.1186/1471-2105-11-516 PMC2964687

[36] Shannon P, Markiel A, Ozier O, Baliga NS, Wang JT, et al (2003) Cytoscape: a software environment for integrated models of biomolecular interaction networks. Genome Res 13: 2498–2504. 14597658 10.1101/gr.1239303 PMC403769

[37] Leduc MS, Blair RH, Verdugo RA, Tsaih SW, Walsh K, et al (2012) Using bioinformatics and systems genetics to dissect HDL-cholesterol genetics in an MRL/MpJ x SM/J intercross. J Lipid Res 53: 1163–1175. 22498810 10.1194/jlr.M025833 PMC3351823

[38] Akita K, Okuno M, Enya M, Imai S, Moriwaki H, et al (2002) Impaired liver regeneration in mice by lipopolysaccharide via TNF-alpha/kallikrein-mediated activation of latent TGF-beta. Gastroenterology 123: 352–364. 12105863 10.1053/gast.2002.34234

[39] Liu H, Ren H, Spear BT (2011) The mouse alpha-albumin (afamin) promoter is differentially regulated by hepatocyte nuclear factor 1alpha and hepatocyte nuclear factor 1beta. DNA Cell Biol 30: 137–147. 20979532 10.1089/dna.2010.1097 PMC3045788

[40] Voegele AF, Jerkovic L, Wellenzohn B, Eller P, Kronenberg F, et al (2002) Characterization of the vitamin E-binding properties of human plasma afamin. Biochemistry 41: 14532–14538. 12463752 10.1021/bi026513v

[41] Phung N, Pera N, Farrell G, Leclercq I, Hou JY, et al (2009) Pro-oxidant-mediated hepatic fibrosis and effects of antioxidant intervention in murine dietary steatohepatitis. Int J Mol Med 24: 171–180. 19578790 10.3892/ijmm_00000220

[42] Sanyal AJ, Chalasani N, Kowdley KV, McCullough A, Diehl AM, et al (2010) Pioglitazone, vitamin E, or placebo for nonalcoholic steatohepatitis. N Engl J Med 362: 1675–1685. 20427778 10.1056/NEJMoa0907929 PMC2928471

[43] Kim BJ, Lee YS, Lee SY, Park SY, Dieplinger H, et al (2012) Afamin secreted from nonresorbing osteoclasts acts as a chemokine for preosteoblasts via the Akt-signaling pathway. Bone 51: 431–440. 22749887 10.1016/j.bone.2012.06.015

[44] Hintermann E, Bayer M, Pfeilschifter JM, Luster AD, Christen U (2010) CXCL10 promotes liver fibrosis by prevention of NK cell mediated hepatic stellate cell inactivation. J Autoimmun 35: 424–435. 20932719 10.1016/j.jaut.2010.09.003 PMC3855675

[45] Beaven SW, Wroblewski K, Wang J, Hong C, Bensinger S, et al (2011) Liver X receptor signaling is a determinant of stellate cell activation and susceptibility to fibrotic liver disease. Gastroenterology 140: 1052–1062. 21134374 10.1053/j.gastro.2010.11.053 PMC3049833

[46] El-Karef A, Kaito M, Tanaka H, Ikeda K, Nishioka T, et al (2007) Expression of large tenascin-C splice variants by hepatic stellate cells/myofibroblasts in chronic hepatitis C. J Hepatol. 46: 664–673. 10.1016/j.jhep.2006.10.011 17188391

[47] Kalembeyi I, Inada H, Nishiura R, Imanaka-Yoshida K, Sakakura T, et al (2003) Tenascin-C upregulates matrix metalloproteinase-9 in breast cancer cells: direct and synergistic effects with transforming growth factor beta1. Int J Cancer 105: 53–60. 12672030 10.1002/ijc.11037

[48] Hsia HC, Schwarzbauer JE (2005) Meet the tenascins: multifunctional and mysterious. J Biol Chem 280: 26641–26644. 15932878 10.1074/jbc.R500005200

[49] Forsberg E, Hirsch E, Frohlich L, Meyer M, Ekblom P, et al (1996) Skin wounds and severed nerves heal normally in mice lacking tenascin-C. Proc Natl Acad Sci USA 93: 6594–6599. 8692862 10.1073/pnas.93.13.6594 PMC39070

[50] Midwood K, Sacre S, Piccinini AM, Inglis J, Trebaul A, et al (2009) Tenascin-C is an endogenous activator of Toll-like receptor 4 that is essential for maintaining inflammation in arthritic joint disease. Nat Med 15: 774–780. 19561617 10.1038/nm.1987

[51] Seki E, De Minicis S, Osterreicher CH, Kluwe J, Osawa Y, et al (2007) TLR4 enhances TGF-beta signaling and hepatic fibrosis. Nat Med 13: 1324–1332. 17952090 10.1038/nm1663

[52] Anstee QM, Darlay R, Leathart J, Liu Y-L, Tordjman J, et al (2013) A candidate-gene approach to validation of genetic modifier associations using a large cohort with histologically characterised non-alcoholic fatty liver disease. J Hepatol 58 46: 104 (Abstract)..

[53] Sookoian S, Pirola CJ (2011) Meta-analysis of the influence of I148M variant of patatin-like phospholipase domain containing 3 gene (PNPLA3) on the susceptibility and histological severity of nonalcoholic fatty liver disease. Hepatology 53: 1883–1894. 21381068 10.1002/hep.24283

