# Supplementary figures and images for: Systems Genetics of Liver Fibrosis: Identification of Fibrogenic and Expression Quantitative Trait Loci in the BXD Murine Reference Population
Source: PLoS One. 2014 Feb 28;9(2):e89279. doi: 10.1371/journal.pone.0089279 (PMC3938463; doi:10.1371/journal.pone.0089279)

Figure S1

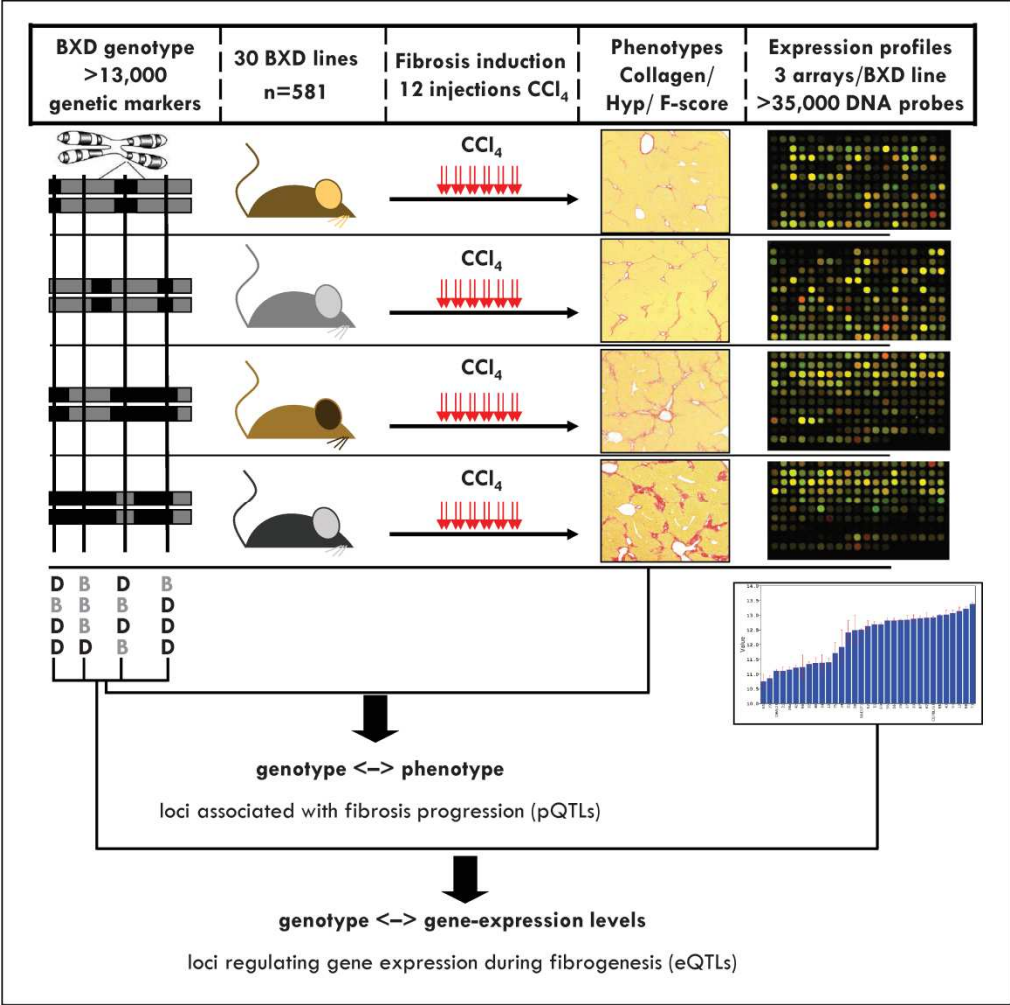

Figure S2

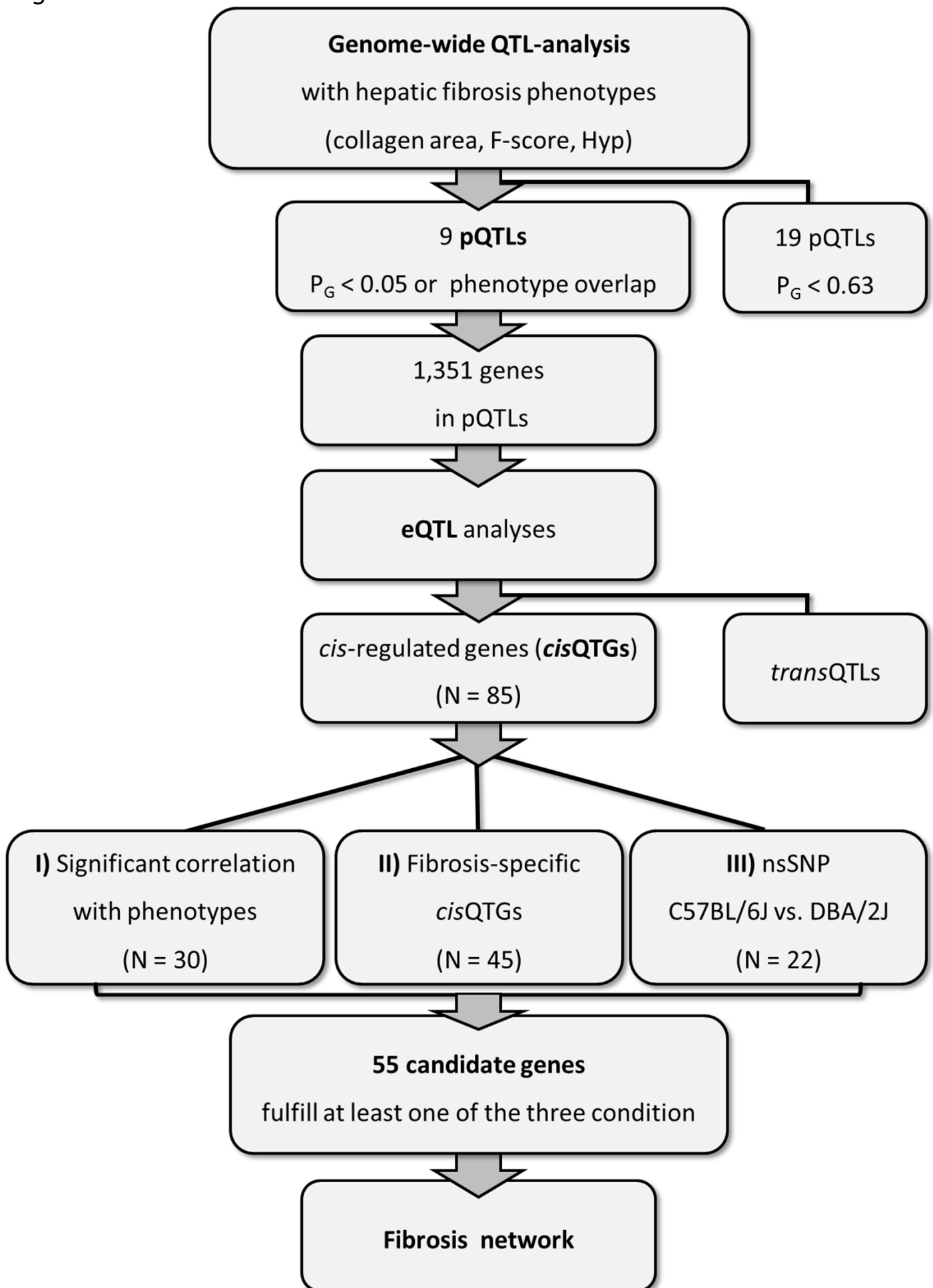

Figure S3

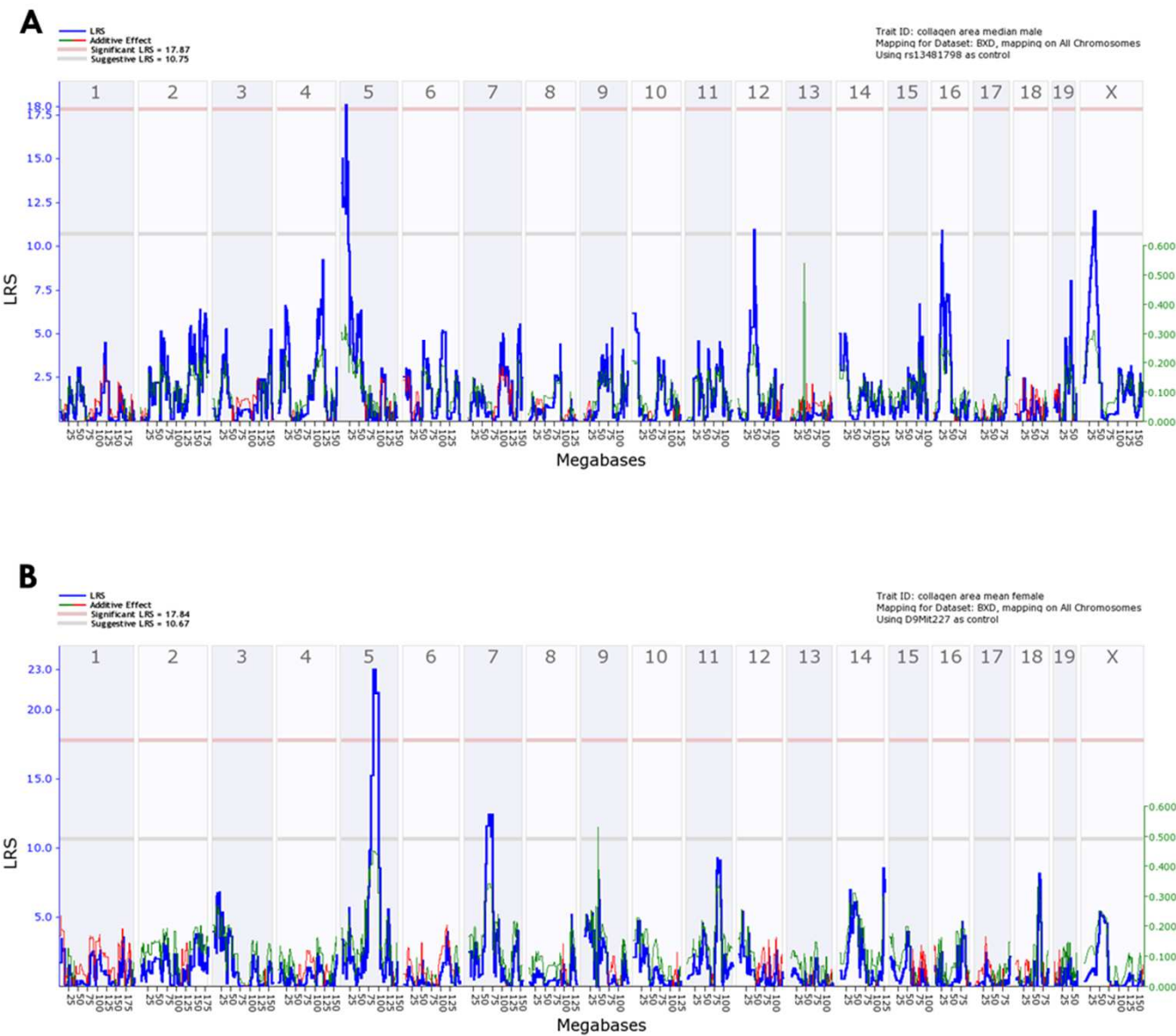

Figure S3

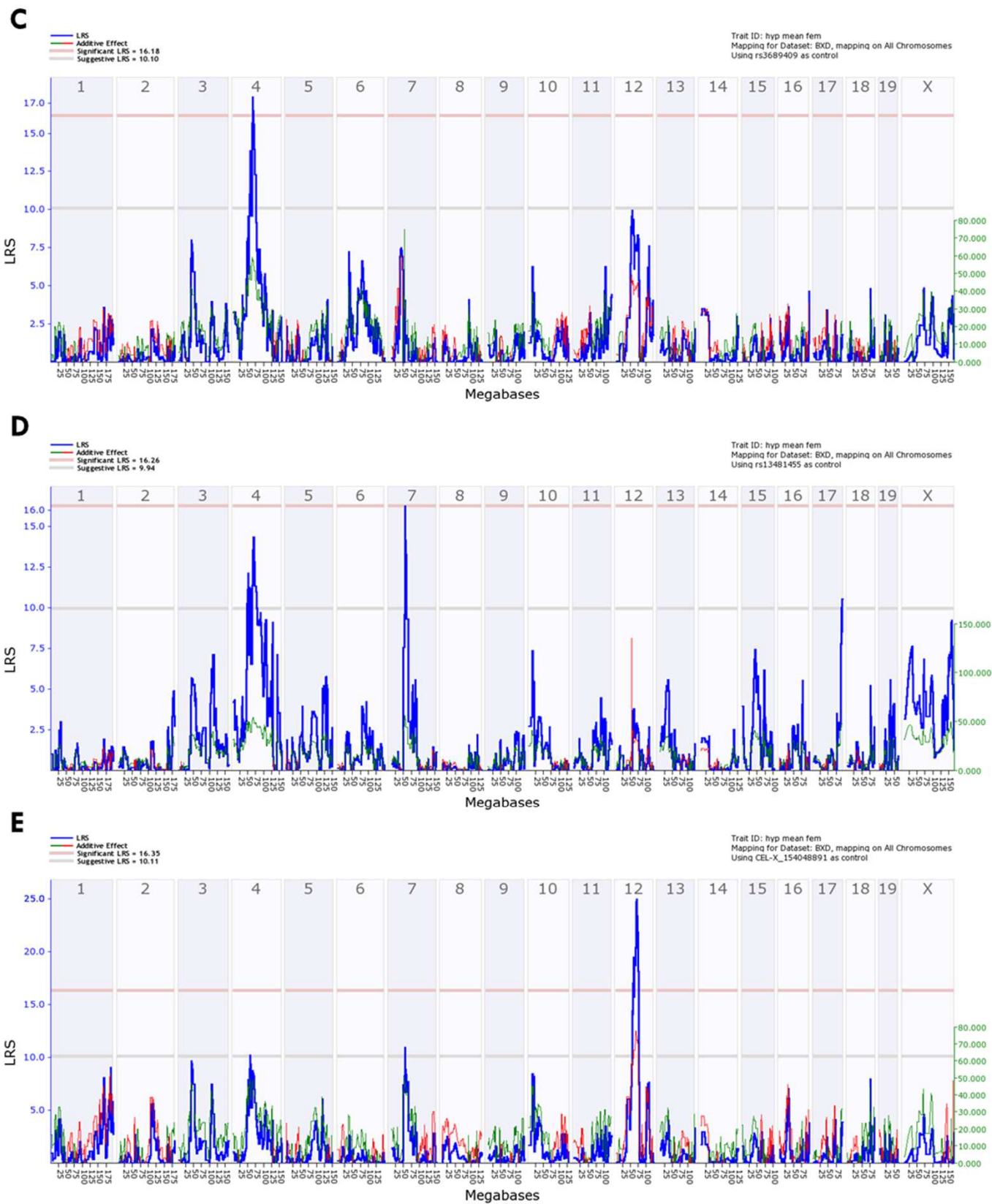

Figure S3

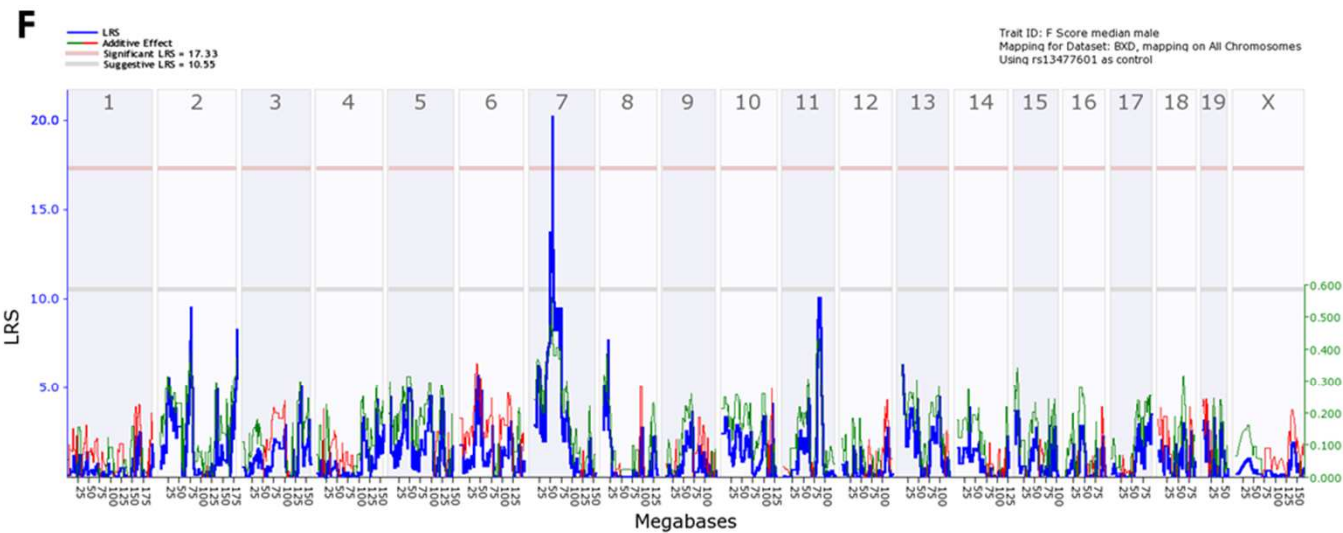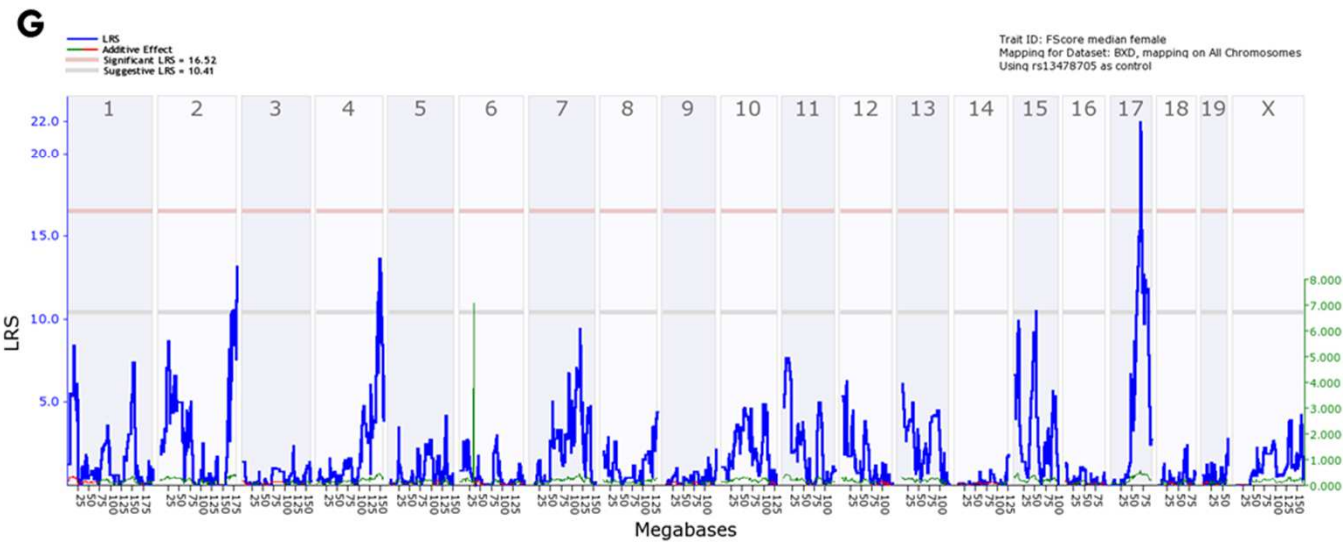

Figure S4

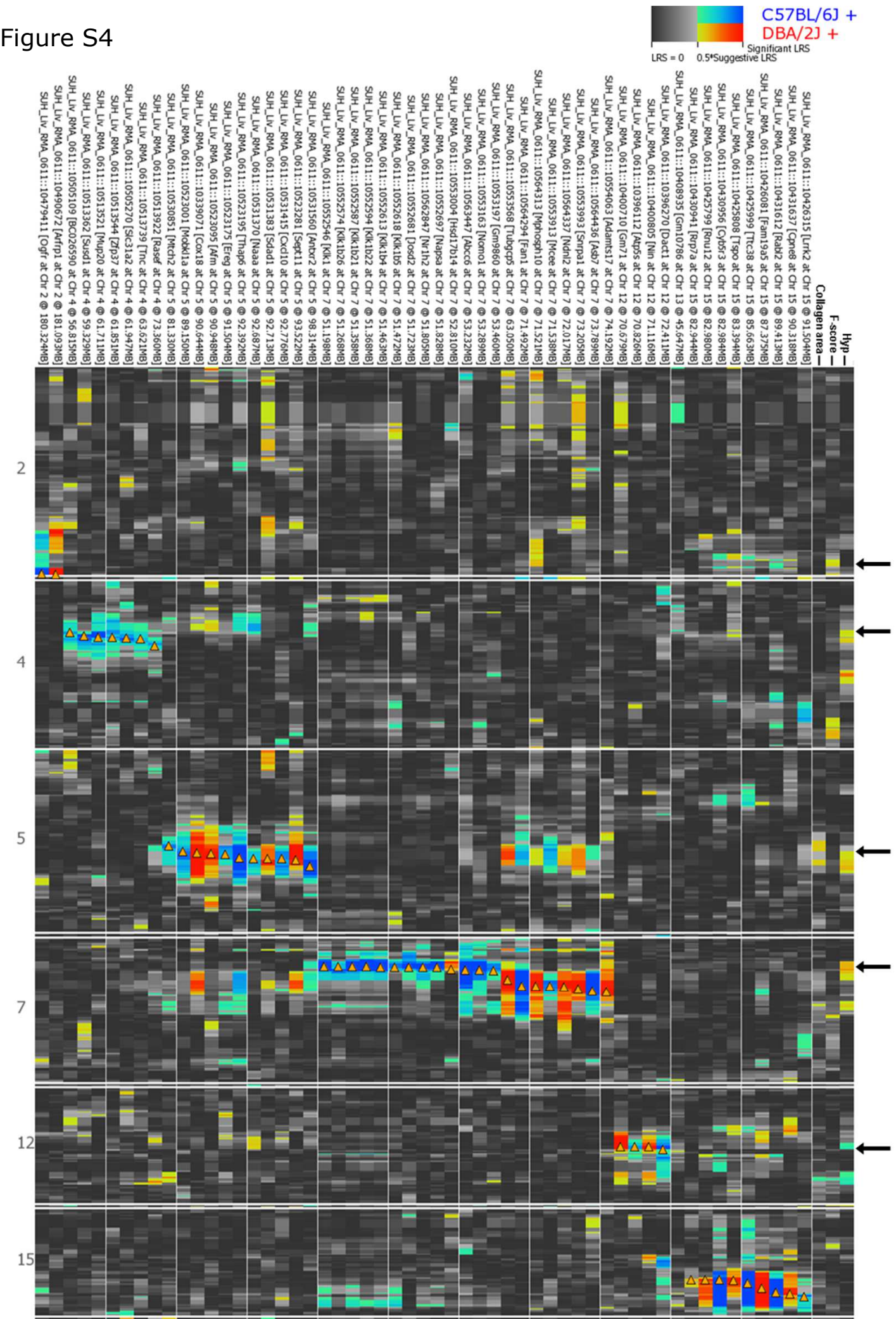

Supplement: File S1 — Figure S1, Graphical overview of the experimental setup for the integrative analysis of pQTLs and eQTLs in the BXD murine reference panel. Abbreviations: BXD, recombinant inbred lines based on parental strains C57BL/6J and DBA/2J; CCl4, carbon tetrachloride; DNA, deoxyribonucleic acid; eQTL, expression quantitative trait locus; F-score, fibrosis score; Hyp, hydroxyproline; n, number; pQTL, phenotypic quantitative trait locus. Figure S2, Study design and strategy for the selection of candidate genes. Genome-wide association studies of CCl4 treated BXD lines identified phenotype-associated QTLs (pQTLs). The 1,351 genes located in significant pQTLs were investigated further by eQTL analyses (see Methods). This allowed the differentiation of local (cis-) or distant (trans)-regulation of gene expression. Cis-regulated genes (cisQTGs) underwent the following three selection steps to refine the list of candidate genes: I) cisQTGs with significant correlation with fibrosis phenotypes; II) fibrosis-specific cisQTGs that show differential regulation between basal state and after the induction of fibrosis; and III) cisQTGs with non-synonymous (ns) SNPs segregating in strains C57BL/6J and DBA/2J. cisQTGs complying with one of the three criteria were considered as creedal candidate genes. In total, 55 candidate genes were included into the fibrosis network. Abbreviations: cisQTGs, cis-regulated genes; eQTL, expression quantitative trait locus; F-score, fibrosis score; Hyp, hydroxyproline; n, number; nsSNP non-synonymous single nucleotide polymorphism; pQTL, phenotypic quantitative trait locus; PG, genome-wide p-value. Figure S3, Single QTL scans identifying significant loci for each fibrosis phenotype. Legend on top left: empirical genome-wide significance thresholds of LRS values, significant (pink line), suggestive (grey line); additive allele effect, DBA/2J alleles (green), C57BL/6J alleles (red). Traits: (A–B) collagen area, (C–E) hepatic collagen (Hyp) concentration and (F- [file pone.0089279.s001.pdf]
