# Supplementary material for: Systems Genetics of Liver Fibrosis: Identification of Fibrogenic and Expression Quantitative Trait Loci in the BXD Murine Reference Population
Source: PLoS One. 2014 Feb 28;9(2):e89279. doi: 10.1371/journal.pone.0089279 (PMC3938463; doi:10.1371/journal.pone.0089279)
Supplement: File S2 — Table S1, Chromosomal regions of pQTLs determined by single QTL scans and CIM. Table S2, Overlapping pQTL regions for different fibrosis phenotypes determined by single QTL scans and CIM. Table S3, Candidate genes of hepatic fibrogenesis. (PDF) [file pone.0089279.s002.pdf]

Table S1: Chromosomal regions of pQTLs determined by single QTL scans and CIM

Phenotype	pQTL (Chr)	LRS (max)	SNP (max)	1.5 LOD support interval (Mb)	Additive allele effect (-) C57BL/6J / (+) DBA/2J	Dataset
Collagen area	2	14.0	rs638488 - rs3664044	167.7 - 179.3	0.303	male
Collagen area	3	12.5	rs13476999 - mCV24211562	12.0 - 20.6	0.331	female
Collagen area	3	15.0	rs13477485 - rs13477494	146.1 - 157.0	0.320	female
Collagen area	5*	18.1	mCV23582150 - rs6392739	3.1 - 20.1	0.323	male/both
Collagen area	5*	23.1	rs3678577 - rs6167407	85.1 - 97.9	0.451	female/both
Collagen area	7	12.5	gnf07.050.858 - rs6166250	55.3 - 74.2	0.340	female
Collagen area	7	12.5	rs13479559 - rs3659292	141.7 - 149.0	0.302	male
Collagen area	9	14.4	D9Mit227 - rs13480169	41.1 - 46.6	-0.350	female
Collagen area	13	16.7	rs6411274 - rs3682400	45.5 - 51.7	0.251	male/both
Collagen area	15	13.9	rs3710055 - rs13482702	82.3 - 95.8	0.265	male
Collagen area	19	13.3	rs13483649 - rs8257607	36.2 - 52.2	0.288	male
Collagen area	X	17.1	gnfX.023.534 - gnfX.026.801	25.2 - 41.9	0.357	male
Hyp	4*	17.4	rs6254381 - rs13477745	55.1 - 73.9	58.957	female/both
Hyp	7*	16.3	rs3703247 - rs8255275	52.8 - 56.7	56.761	female/both
Hyp	5	14.4	rs13478413 - rs6228198	82.8 - 103.9	49.673	female/both
Hyp	12*	25.0	rs3716547 -rs13481511	60.5 - 73.3	-77.257	female/both
Hyp	X	12.9	CEL-X_154048891	145.8 - 162.6	53.563	female
F-score	2	13.3	rs3719468	74.9 - 76.8	0.365	male/both
F-score	2	15.2	rs6305540 - rs6402916	174.5 - 181.5	0.395	male/female/ both
F-score	4	13.7	rs4224919 - rs3669806	139.1 - 152.7	0.442	female
F-score	7*	20.3	rs3703247 - rs8255275	48.2 - 53.7	0.562	male/both
F-score	8	12.8	rs3699325 - rs13479628	9.13 - 18.42	0.415	male
F-score	11	12.1	rs13481150 - rs3688955	79.8 - 93.5	0.372	male/both
F-score	13	16.7	rs3688207 - rs429721	44.2 - 52.7	0.430	male/both
F-score	15	12.9	rs13482436 - rs4139555	9.4 - 15.3	0.396	female
F-score	15	16.7	rs13482547 - rs3692040	44.2 - 51.7	0.406	female
F-score	15	13.8	rs13482723 - 3664692	92.8 - 95.9	0.395	male
F-score	17*	22.0	rs13483077 - rs13483081	64.9 - 71.1	0.516	female

Abbreviations and definitions:

pQTL (chr): chromosomal position of phenotype linked quantitative trait locus; (*): indicates a QTL significantly associated with the respective phenotype; **LRS (max):** likelihood ratio statistic, maximum association between genotype and phenotype variation; **SNP (max):** single nucleotide polymorphism with maximum LRS in QTL region; **1.5 LOD support interval (Mb):** chromosomal region in Megabases spanning QTL position; **Additive allele effect:** estimate of a change in the average phenotype by substitution of one parental allele by another at a given marker position; **(-)** values indicate an increase of phenotype by C57BL/6J allele, **(+)** values indicate an increase of phenotype by DBA/2J allele. **Dataset**: dataset in which the QTL was identified; **Hyp**: hydroxyproline; **CIM**: composite interval mapping.

Table S2: Overlapping pQTL regions for different fibrosis phenotypes determined by single QTL scans and CIM

pQTL (Chr)	Phenotype	LRS (max)	SNP (max)	1.5 LOD support interval (Mb)	Additive allele effect (-) C57BL/6J (+) DBA/2J	Dataset
2	Collagen area	14.0	rs638488-rs3664044	167.7 - 179.3	0.303	male
	F-score	15.2	rs6305540-rs6402916	174.5 - 181.5	0.395	male/female/both
5	Hyp	14.4	rs13478413-rs6228198	82.8 - 103.9	49.673	female/both
	Collagen area	23.1	rs3678577-rs6167407	85.1 - 97.9	0.451	female/both
7	F-score	20.3	rs3703247-rs8255275	48.2 - 53.7	0.562	male/both
	Hyp	16.3	rs3703247-rs8255275	52.8 - 56.7	56.761	female/both
	Collagen area	12.5	gnf07.050.858-rs6166250	55.3 - 74.2	0.34	female
13	F-score	16.7	rs3688207-rs429721	44.2 - 52.7	0.43	male/both
	Collagen area	16.7	rs6411274-rs3682400	45.5 - 51.7	0.251	male/both
15	Collagen area	13.9	rs3710055-rs13482702	82.3 - 95.8	0.265	male
	F-score	13.8	rs13482723-3664692	92.8 - 95.9	0.395	male

Abbreviations and definitions:

pQTL (chr): chromosomal position of quantitative trait locus; **LRS (max):** likelihood ratio statistic, maximum association between genotype and phenotype variation; **SNP (max):** single nucleotide polymorphism with maximum LRS in QTL region; **1.5 LOD support interval (Mb):** chromosomal region in Megabases spanning QTL position; **Additive allele effect:** estimate of a change in the average phenotype by substitution of one parental allele by another at a given marker position; **(-)** values indicate an increase of phenotype by C57BL/6J allele, **(+)** values an increase of phenotype by DBA/2J allele; **Dataset:** dataset in which the QTL was identified; **Hyp**: hydroxyproline; **CIM**: composite interval mapping.

Table S3: Candidate genes of hepatic fibrogenesis

*cis*QTL				Gene regulation				Gene to phenotype correlation			Selection criteria		
	**pQTL (Chr)**	**Gene symbol**	**Description**	**Location (Chr @ Mb)**	**Mean expression**	**Max LRS**	**Max LRS location (Chr @ Mb)**	**Collagen area**	**Hyp**	**F-score**	**1.Correlated with phenotype**	**2. Fibrosis specific regulation**	**3.nsSNP**
1	2	*Ogfr*	opioid growth factor receptor	Chr 2 @ 180.3	10.1	23.6	Chr2: 180.8	-0.027	-0.222	-0.158			●
2		*Arfrp1*	ADP-ribosylation factor related protein 1	Chr 2 @ 181.1	9.1	29.8	Chr2: 180.8	0.116	0.216	0.416*	●		
3	4	*BC026590*	cDNA sequence BC026590	Chr 4 @ 56.8	9.3	15.5	Chr4: 56.5	-0.007	-0.383*	-0.181	●	●	
4		*Susd1*	sushi domain containing 1	Chr 4 @ 59.3	8.6	17.4	Chr4: 58.4	0.002	-0.503*	0.051	●	●	●
5		*Mup20*	major urinary protein 20	Chr 4 @ 61.7	13.0	16.8	Chr4: 58.4	-0.075	-0.242	-0.001		●	
6		*Zfp37*	zinc finger protein 37	Chr 4 @ 61.9	7.3	15.7	Chr4: 62.4	-0.059	-0.188	-0.122		●	●
7		*Slc31a2*	solute carrier family 31, meer 2	Chr 4 @ 61.9	10.8	20.3	Chr4: 63.3	-0.005	-0.391*	-0.311	●	●	
8		*Tnc*	tenascin C	Chr 4 @ 63.6	8.0	13.2	Chr4: 65.6	0.120	-0.332	-0.291		●	
9		*Rasef*	RAS and EF hand domain containing	Chr 4 @ 73.4	6.4	13.7	Chr4: 69.8	-0.180	-0.414*	0.039	●	●	
10	5	*Mtch2*	mitochondrial carrier homolog 2 (C.elegans)	Chr 5 @ 81.3	12.0	16.5	Chr5: 82.8	-0.482*	-0.317	-0.234	●	●	
11		*Mobkl1a*	MOB1, Mps One Binder kinase activator-like 1A (yeast)	Chr 5 @ 89.1	10.4	12.1	Chr5: 90.5	-0.079	-0.176	0.334		●	
12		*Cox18*	COX18 cytochrome c oxidase assely homolog (complex iV assely)	Chr 5 @ 90.6	11.8	59.7	Chr5: 94.2	0.402*	0.397	0.127	●		●
13		*Afm*	afamin	Chr 5 @ 90.9	11.3	15.8	Chr5: 90.5	0.257	0.429*	0.336	●	●	●
14		*Ereg*	epiregulin	Chr 5 @ 91.5	7.2	13.0	Chr5: 82.8	-0.278	-0.405*	-0.087	●	●	
15		*Thap6*	THAP domain containing 6	Chr 5 @ 92.4	8.6	54.3	Chr5: 90.5	-0.356	-0.424*	-0.105	●	●	
16		*Naaa*	N-acylethanolamine acid amidase	Chr 5 @ 92.7	9.5	13.8	Chr5: 94.2	-0.075	-0.425*	-0.389	●		●
17		*Sdad1*	SDA1 domain containing 1	Chr 5 @ 92.7	7.6	13.3	Chr5: 94.2	0.369*	0.247	0.249	●		●
18		*Cxcl10*	chemokine (C-X-C motif) ligand 10	Chr 5 @ 92.8	9.7	13.7	Chr5: 98.2	-0.115	-0.070	0.045		●	
19		*Sept11*	septin 11	Chr 5 @ 93.5	10.2	21.5	Chr5: 85.1	0.199	0.068	-0.222		●	
20		*Antxr2*	anthrax toxin receptor 2	Chr 5 @ 98.3	10.4	24.7	Chr5: 98.2	-0.056	-0.527*	-0.165	●		
21	7	*Klk1*	kallikrein 1	Chr 7 @ 51.2	8.6	42.2	Chr7: 48.2	-0.047	-0.438*	-0.169	●	●	
22		*Klk1b26*	kallikrein 1-related petidase b26	Chr 7 @ 51.3	8.0	15.2	Chr7: 53.3	-0.233	-0.413*	-0.086	●	●	●
23		*Klk1b21*	kallikrein 1-related peptidase b21	Chr 7 @ 51.4	7.8	39.6	Chr7: 48.2	-0.017	-0.449*	-0.111	●	●	●
24		*Klk1b22*	kallikrein 1-related peptidase b22	Chr 7 @ 51.4	6.3	38.4	Chr7: 48.2	-0.027	-0.361*	-0.032	●	●	●
25		*Klk1b4*	kallikrein 1-related pepidase b4	Chr 7 @ 51.5	9.5	39.0	Chr7: 48.2	-0.085	-0.473*	-0.181	●		
26		*Klk1b5*	kallikrein 1-related peptidase b5	Chr 7 @ 51.5	6.8	18.4	Chr7: 48.2	-0.109	-0.370*	-0.305	●	●	●
27		*Josd2*	Josephin domain containing 2	Chr 7 @ 51.7	9.6	16.1	Chr7: 48.2	-0.183	-0.165*	-0.141		●	
28		*Nr1h2*	nuclear receptor subfamily 1, group H, meer 2	Chr 7 @ 51.8	10.6	23.4	Chr7: 52.8	0.031	-0.207	0.066		●	
29		*Napsa*	napsin A aspartic peptidase	Chr 7 @ 51.8	8.0	19.7	Chr7: 48.2	0.069	-0.489*	-0.271	●	●	●
30		*Hsd17b14*	hydroxysteroid (17-beta) dehydrogenase 14	Chr 7 @ 52.8	7.4	17.9	Chr7: 47.6	0.114	0.424*	0.265	●	●	●
31		*Abcc6*	ATP-binding cassette, sub-family C (CFTR/MRP), meer 6	Chr 7 @ 53.2	10.0	26.3	Chr7: 53.3	-0.226	-0.316	-0.276	●		●
32		*Nomo1*	nodal modulator 1	Chr 7 @ 53.3	10.5	23.0	Chr7: 48.2	-0.110	-0.483*	-0.219	●	●	●
33		*Gm9860*	predicted gene 9860	Chr7 @ 53.5	7.2	18.2	Chr7: 52.8	-0.177	-0.360*	-0.204	●	●	
34		*Tubgcp5*	tubulin, gamma complex associated protein 5	Chr 7 @ 63.0	8.1	32.3	Chr7: 56.7	0.209	0.321	0.041		●	●
35		*Fan1*	FANCD2/FANCI-associated nuclease 1	Chr 7 @ 71.5	8.0	48.9	Chr7: 63.8	-0.363*	-0.245	-0.204	●	●	●
36		*Mphosph10*	M-phase phosphoprotein 10 (U3 small nucleolar ribonucleoprotein)	Chr 7 @ 71.5	8.3	22.7	Chr7: 63.8	0.249	0.184	0.351			●
37		*Mcee*	methylmalonyl CoA epimerase	Chr 7 @ 71.5	12.5	15.1	Chr7: 63.8	-0.492*	0.010	-0.047	●	●	
38		*Ndnl2*	necdin-like 2	Chr 7 @ 72.0	9.1	20.0	Chr7: 63.8	0.396*	0.258	0.315	●		
39		*Snrpa1*	small nuclear ribonucleoprotein polypeptide A'	Chr 7 @ 73.2	9.3	25.0	Chr7: 73.3	0.263	0.192	0.030		●	
40		*Asb7*	ankyrin repeat and SOCS box-containing 7	Chr 7 @ 73.8	8.3	27.2	Chr7: 73.3	-0.136	-0.268	-0.053		●	●
41		*Adamts17*	a disintegrin-like and metallopeptidase (reprolysin type) with throospondin type 1 motif, 17	Chr 7 @ 74.2	7.2	17.8	Chr7: 73.3	0.171	0.132	0.116		●	●
42	12	*Gm71*	predicted gene 71	Chr 12 @ 70.7	8.5	34.5	Chr12: 70.7	-0.217	-0.360	-0.025	●	●	
43		*Atp5s*	ATP synthase, H+ transporting, mitochondrial F0 complex, subunit s	Chr 12 @ 70.8	7.9	20.3	Chr12: 70.7	0.090	0.220	0.227		●	
44		*Nin*	ninein	Chr 12 @ 71.1	8.5	18.2	Chr12: 70.7	-0.180	-0.498*	-0.290	●	●	●
45		*Dact1*	dapper homolog 1, antagonist of beta-catenin (xenopus)	Chr 12 @ 72.4	8.0	15.2	Chr12: 70.7	0.249	0.244	0.186		●	●
46	13	*Gm10786*	predicted gene 10786	Chr 13 @ 45.6	9.4	16.0	Chr13: 45.5	0.146	0.009	-0.038		●	
47	15	*Rrp7a*	ribosomal RNA processing 7 homolog A (S. cerevisiae)	Chr 15 @ 82.9	9.7	12.8	Chr15: 82.9	-0.119	-0.015	0.270		●	
48		*Rnu12*	RNA U12, small nuclear	Chr 15 @ 83.0	7.5	16.6	Chr15: 91.7	-0.222	0.128	0.035		●	
49		*Cyb5r3*	NADH-cytochrome b5 reductase 3 (diaphorase-1, methemoglobinemia)	Chr 15 @ 83.0	13.5	60.8	Chr15: 82.9	0.102	-0.112	-0.221		●	
50		*Tspo*	translocator protein	Chr 15 @ 83.4	10.8	18.2	Chr15: 82.9	-0.263	-0.019	0.056		●	
51		*Ttc38*	tetratricopeptide repeat domain 38	Chr 15 @ 85.7	10.4	56.6	Chr15: 82.9	0.289	0.128	-0.011		●	
52		*Fam19a5*	family with sequence similarity 19, meer A5	Chr 15 @ 87.4	8.3	23.5	Chr15: 87.8	-0.329	-0.139	0.144		●	
53		*Rabl2*	RAB, meer of RAS oncogene family-like 2	Chr 15 @ 89.4	8.2	52.8	Chr15: 87.8	0.167	-0.060	-0.201		●	
54		*Cpne8*	copine VIII	Chr 15 @ 90.3	7.8	21.4	Chr15: 90.6	-0.024	-0.191	-0.048		●	
55		*Lrrk2*	leucine-rich repeat kinase 2	Chr 15 @ 91.5	7.9	17.2	Chr15: 90.8	0.295	0.208	-0.402*	●	●	

Abbreviations and definitions:

***cis*QTG**: genes located in the pQTL region (regulated by markers within a 10 Mb distance) with LRS ≥ 12.0; **Location (Chr @ Mb)**: Chromosomal position in Megabases of candidate gene; **Mean expression**: mean expression value determined across all BXD lines; **LRS (max):** likelihood ratio statistic, maximum association detected in pQTL analysis; **Max LRS location (Chr @ Mb):** Chromosomal position of regulatory genetic marker with maximum association to *cis*QTG; **Gene to phenotype correlation**: Pearson correlation coefficient r with a **(-)** negative or **(+)** positive correlation of the *cis*QTG to fibrosis phenotypes; (*): indicate a significant correlation ($p < 0.05$); **Selection criteria:** (●) indicates that the gene fulfills the following selection criteria for candidate genes: 1) significant correlation to phenotype, 2) differentially regulated during fibrosis as compared with healthy animals, or 3) non-synonymous single nucleotide polymorphism (**nsSNP**) in coding region of the gene.
